# Carbon-Based Composites as Electrocatalysts for Oxygen Evolution Reaction in Alkaline Media

**DOI:** 10.3390/ma14174984

**Published:** 2021-08-31

**Authors:** Paweł Stelmachowski, Joanna Duch, David Sebastián, María Jesús Lázaro, Andrzej Kotarba

**Affiliations:** 1 Faculty of Chemistry, Jagiellonian University, Gronostajowa 2, 30-387 Krakow, Poland; ak@uj.edu.pl; 2 Department of Bioinorganic Chemistry, Faculty of Chemistry, University of Gdańsk, Wita Stwosza 63, 80-308 Gdańsk, Poland; joanna.duch@ug.edu.pl; 3 Instituto de Carboquímica (CSIC), Miguel Luesma Castán 4, 50018 Zaragoza, Spain; dsebastian@icb.csic.es (D.S.); mlazaro@icb.csic.es (M.J.L.)

**Keywords:** water electrolysis, hydrogen production, energy conversion, carbon composites

## Abstract

This review paper presents the most recent research progress on carbon-based composite electrocatalysts for the oxygen evolution reaction (OER), which are of interest for application in low temperature water electrolyzers for hydrogen production. The reviewed materials are primarily investigated as active and stable replacements aimed at lowering the cost of the metal electrocatalysts in liquid alkaline electrolyzers as well as potential electrocatalysts for an emerging technology like alkaline exchange membrane (AEM) electrolyzers. Low temperature electrolyzer technologies are first briefly introduced and the challenges thereof are presented. The non-carbon electrocatalysts are briefly overviewed, with an emphasis on the modes of action of different active phases. The main part of the review focuses on the role of carbon–metal compound active phase interfaces with an emphasis on the synergistic and additive effects. The procedures of carbon oxidative pretreatment and an overview of metal-free carbon catalysts for OER are presented. Then, the successful synthesis protocols of composite materials are presented with a discussion on the specific catalytic activity of carbon composites with metal hydroxides/oxyhydroxides/oxides, chalcogenides, nitrides and phosphides. Finally, a summary and outlook on carbon-based composites for low temperature water electrolysis are presented.

## 1. Introduction

The world economy is expanding continuously, and energy demand increases likewise. It is predicted that the energy consumption will double by 2050 and triple by 2100 compared to the year 2001 [1,2]. The concomitant increase in CO_2_ emissions makes the current situation critical, resulting in the increased implementation of new policies for environmental protection. In addition, the limited fossil fuel reserves and their uneven distribution on the Earth call for a more distributed way of energy conversion and storage. Therefore, one of the greatest challenges for humanity in the 21st century is the development of renewable energy systems [1]. Within this area, the aspects of efficient conversion and energy storage in a safe, effective, and scalable way hold the key to commercial applications [3,4,5,6].

Currently, large amounts of energy are stored through the pumped hydro energy storage systems. However, its biggest disadvantage is its dependence on specific geographical features for installation and political environment [4]. In the meantime, hydrogen is considered one of the energy vectors that will play a key role in future mobile energy conversion devices [6,7,8,9]. Hydrogen is very abundant in nature but is bound in the form of H_2_O or C_x_H_y_. To become a sustainable energy carrier, the production process of hydrogen has to use renewable energy sources (sun, wind, hydropower) and the substrate itself has to be renewable (H_2_O).

Water electrolysis using renewable energy sources and water as a substrate may be an answer to the problem of sustainable hydrogen production. It is also a fascinating subject for studies from the fundamental point of view as the reaction itself is very complex, as are the reaction conditions. The oxidation of oxygen atoms from water releases the protons and electrons required for the formation of hydrogen gas. However, water oxidation, whether in acidic or alkaline conditions, suffers from the kinetic limitations of the oxygen evolution reaction (OER) at the anode rather than by the limitations associated with the hydrogen evolution reaction (HER) at the cathode. The reason is mostly due to the four-electron transfer process of the OER compared to the two-electron transfer step of the HER [7]. At the same time, for practical applications, the architecture of the electrode interface plays an equally important role, since a triple-phase boundary of gas, liquid electrolyte, and solid electrocatalyst is the scene where the water electrolysis takes place [10]. 

**Figure 1:**
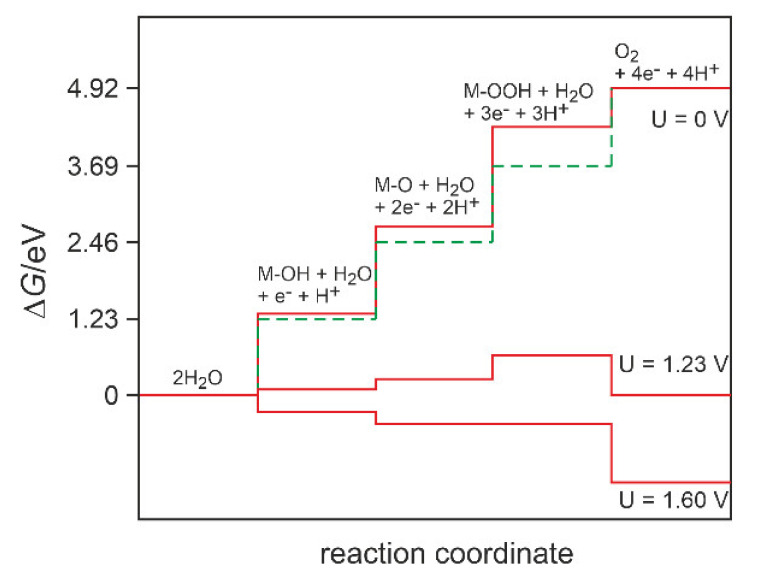
Energetics of OER over RuO_2_. Red, solid lines depict the Gibbs free energies of the reaction intermediates at three different external potentials *U* = 0 V, *U* = 1.23 V, and *U* = 1.60 V. Only at 1.60 V are all the reaction steps thermodynamically favorable. Green, dashed line depicts the ideal 1.23 V barrier for all of the reaction steps. Figure adapted from [12].

The equilibrium redox potential (*E*_r_) for the splitting of a water molecule is 1.23 V. For practical yields in electrolysis cells, a higher voltage is required. This additional voltage, above 1.23 V, is called the overpotential (*η*). From the mechanistic point of view, the most popular proposed reaction schemes involve the coupling of electron and proton transfer in every step. If this coupling was always present in the oxygen evolution reaction pathway, it would put severe limitations on the optimization of the catalysts. In such a case, the energy barrier for each of the electron transfer steps would be different, and thus at least one would be higher than 1.23 V and the thermodynamic potential of the overall reaction could not be attained (Figure 1). However, highly active OER catalysts can be designed as multi-component materials, where the proposed linear correlation of the binding energies of intermediates (scaling relations) are valid no more, and other mechanisms may come into play [11]. Nonetheless, high catalytic activity is only one of the requirements for a practical application of an OER catalyst in water splitting devices since the catalytic materials are also required to exhibit long-term stability.

**Figure 2:**
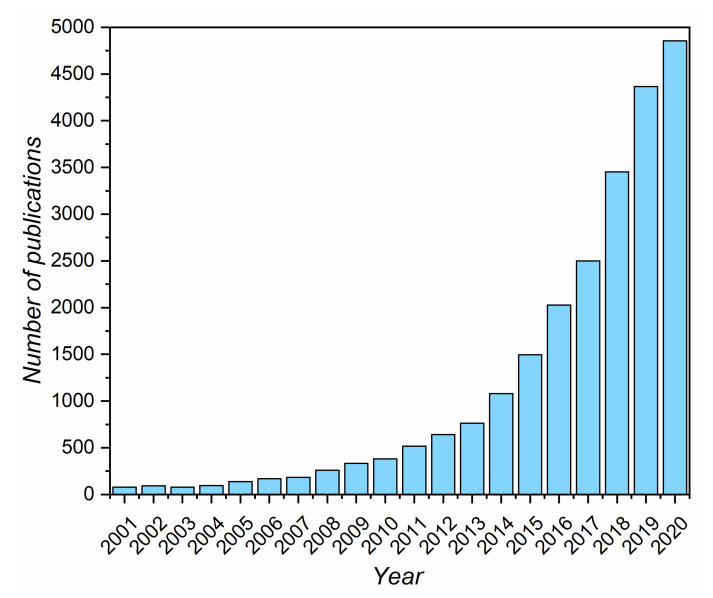
Scopus search results on TITLE-ABS-KEY ((“water splitting” OR (water AND electrolysis) OR “oxygen evolution” OR oer OR “water oxidation”) AND (catalyst OR catalytic OR electrocatalyst OR electrocatalytic)), July 2021.

From a scientific point of view, catalytic water electrolysis has attracted considerably increased attention in the last two decades (Figure 2). 

Three main water splitting processes are studied to the largest extent: liquid alkaline water electrolysis (AWE), proton exchange membrane (PEM) electrolysis, and anion exchange membrane (AEM) electrolysis. Two former ones are already commercial technologies, the liquid alkaline with an over 50-year history [13], while the AEM is still an emerging technology [9]. Electrode reactions taking place in liquid alkaline and anion exchange membrane electrolyzers can be written as:

Anode: 4OH^−^ ⇄ 2H_2_O + O_2_ + 4e^−^ (1)

Cathode: 4H_2_O + 4e^−^ ⇄ 2H_2_ + 4OH^−^ (2)

For PEM electrolyzers, the electrode reactions are:

Anode: 2H_2_O ⇄ O_2_ + 4H^+^ + 4e^−^ (3)

Cathode: 4H^+^ + 4e^−^ ⇄ 2H_2_ (4)

Other related popular research areas include solid oxide electrolysis (SOE) [14] and direct photoelectrocatalytic (PEC) water splitting [15]. Oxygen evolution is an important reaction not only in the water electrolysis application, but also in reversible fuel cells, and metal–air batteries, and thus it is anticipated that OER will play a vital role in the design of efficient energy conversion and storage devices [16].

## 2. Water Splitting Systems

There are many excellent review papers on hydrogen generation through water splitting, which the reader is encouraged to read. These reports include recent progress reports on low-cost hydrogen production with anion exchange membrane electrolyzers [17], including hydroxide exchange membranes, with a tentative cost analysis [18]. The advances in alkaline water electrolyzers are described in [4], while solar-to-hydrogen energy conversion based on water splitting is covered in [19].

**Figure 3:**
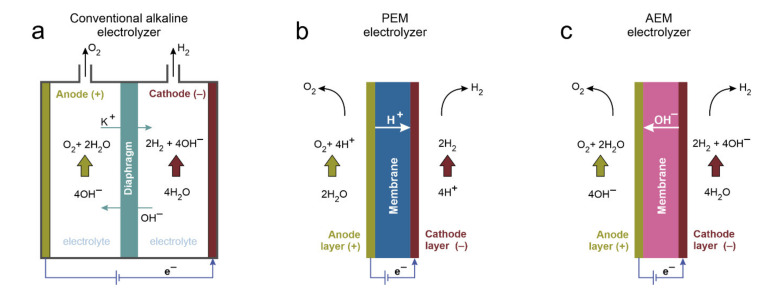
Schematic diagrams of electrolyzers for low temperature water splitting. Commercially available technologies: (**a**) liquid KOH and (**b**) proton exchange membrane (PEM) electrolyzers. Development stage technology: (**c**) anion exchange membrane (AEM) electrolysis.

Due to the high abundance of original research papers related to the topic of water splitting (Figure 2), the presented state-of-the-art knowledge is mostly based on recent publications, and the description is not intended to be comprehensive. The conditions for water splitting systems, and thus the most active phases, differ between liquid alkaline water, proton exchange membrane, and anion exchange membrane electrolysis. Alkaline electrolyzers are characterized by a robust performance, long lifetime, and low cost of the electrode materials, especially anodic, which consist mainly of abundant transition metals (Fe, Ni, Co, Cu, and Mn oxides) and carbon-based materials (nitrogen-doped carbons). AWEs have been commercially employed for a long time now, and they represent a mature technology. In their application, however, the parts of the electrolyzer systems, such as separators and catalysts, that were developed many years ago are still used, while novel alternatives are already present. The main obstacle to be overcome to improve the long-term durability is the high overpotential of the anodic reaction, which causes corrosion of the active phase and support materials. Alkaline water electrolyzers comprise a set of electrodes separated by a diaphragm immersed in an alkaline solution, usually 6 M potassium hydroxide (25 to 30 wt.%) (Figure 3) [7]. Currently, the biggest challenges for AWEs include increasing porosity of the electrodes and catalytic layer to enhance reagent diffusion and gas bubble removal to decrease the ohmic fall, modification of the Ni cathode to increase its durability against the formation of hydride, which results in decreased mechanical stability (hydrogen embrittlement), and improvement of the separators to obtain gasses with greater purity [4]. 

### 2.1. Proton Exchange Membrane Electrolyzers 

PEM electrolyzers, in comparison to AEM systems, are beneficial in terms of lower ohmic losses, higher purity of produced gases, lower overpotentials, higher operating current density, swifter responsiveness, larger range of partial load, and a more compact electrolyzer device. The main disadvantages of proton exchange electrolyzers are due to restrictions as to the anode catalysts, and also other device parts such as separator plates and current collectors since the heavily corrosive environment in combination with high electrode potentials requires high durability of the materials [11]. The development of more robust PEM electrolyzers can be enabled through the designing of more chemically and thermomechanically durable solid polymeric electrolyte membranes, resolving the issue of degradation of anodic collector bipolar plates, developing catalysts to replace Ir (or other noble metals), increasing the purity through decreasing the diffusivity in the solid phase of membranes [4]. The corrosive environment in the PEM cells requires the anodic catalysts to be based on the noble metals, electrochemically oxidized, for which the general trend of activity follows the order Ru > Ir > Rh > Pt > Au [11]. The catalysts’ development focuses on the optimal utilization of noble metals, preferably with atomic efficiency, although Mn-based oxides were recently reported to exhibit promising stability at low potentials [20,21].

### 2.2. Alkaline Exchange Membrane Electrolyzers

AEM electrolysis is an emerging technology. The development of polymeric anion exchange membranes has gained momentum due to their application in alkaline fuel cells. However, they can also be used in water electrolysis systems, offering several benefits such as the use of catalysts based on transition metals instead of noble metal-based materials, use of distilled water (or low concentration alkaline solution) instead of high concentration KOH solution, cheaper substrates for the membrane production (quaternary ammonium ion-exchange-group-containing compounds) in comparison with the Nafion^®^-based membranes, decreased degradation through to interaction with CO_2_ due to lack of metal ions in the AEM structure, and more versatility of the device engineering due to the absence of a corrosive liquid electrolyte. To develop this technology for commercial applications some important issues have to be addressed, such as improving the anionic conductivity of polymers for the membranes, developing improved hydroxyl ion conductivity in ionomers, developing the OER and HER catalysts, deeper characterization of the membrane electrode assemblies from the physical and electrochemical standpoint, and commercialization and industrialization of AEM electrolysis [17].

Recently, Park et al. reported on a Ni/C catalyst alloyed with Co and supported on carbon cloth for an AEM water electrolyzer [22]. The electrocatalyst was applied as the cathode in stack cell (five-cell), demonstrating a performance of 740 mA cm^−2^ at 9.25 V stack and almost 70% efficiency at 440 mA cm^−2^ (150 h). Lindquist et al. investigated an AEM electrolyzer consisting of commercially available electrocatalytic materials (carbon paper as a component of the cathode) that performed below 2 V at 1 A cm^−2^ in pure water [23]. More recent examples of the application of carbon-based materials for AEM electrolyzers can be found in [24,25] Despite a growing number of articles devoted to alkaline exchange membrane electrolyzers, only a small portion of studies show cell performance results [26]. Nevertheless, the available data points to the increasing progress of cell performance achieved in the last decade, giving hope for the development of commercial AEM systems. Nowadays, one of the leading companies working on commercial AEM electrolyzers is Enapter. According to Enapter, their modular devices, using AEM water electrolysis, enable the production of 500 NL h^−1^ of green H_2_ (99.9% purity) [27]. Furthermore, the expansion of AEM technology can be accelerated by the international HydroGEN consortium, which works on the development of advanced water-splitting technologies [24].

Both alkaline water and alkaline exchange membrane electrolyzers can benefit from advancements of similar catalysts for higher efficiency. However, it has to be remembered that the typical results obtained with the rotating disc electrode in a three-electrode cell may be ill-suited to evaluate the catalysts’ oxygen-evolution activity for AEM systems. While the NiFeO_x_H_y_ oxyhydroxides are among the best oxygen evolution reaction catalysts in alkaline electrolytes, they perform poorly in AEM electrolyzers, where pure water is used in combination with the solid alkaline electrolyte. Under AEM electrolyzer conditions, the NiCoO_x_-based catalysts are among the best performing materials. Therefore, different catalysts’ compositions have to be tested in relevant conditions to determine their structure–activity relationships [28].

### 2.3. A Brief Overview of Carbon-Free Catalytic Materials for Oxygen Evolution Reaction in Alkaline Media

In this section, a non-exhaustive review of carbon-free OER catalysts is given to illustrate the most important approaches to date. In Table 1, as well as in subsequent tables, the activity results are reported in terms of overpotential, that is, the deviation of potential from equilibrium (*E*_r_ = 1.23 V vs. reversible hydrogen electrode, RHE) required to cause a given current density (10 mA cm^−2^). Another important parameter is the Tafel slope, representing the variation in overpotential with the logarithmic increase of current, i.e., as well as overpotential, the lower the better. Tafel slope is an indication of the reaction mechanism since it is related to the rate-determining step. In the oxygen evolution, the reaction mechanism in alkaline medium can be described as a series of steps assuming a single-site mechanism (* denotes an active site), as follows (Equations (5)–(9)):

* + OH^−^ ⇌ *OH + e^−^        Tafel slope = 120 mV dec^−1^ (5)

*OH + OH^−^ ⇌ *O + H_2_O + e^−^      Tafel slope = 30 mV dec^−1^ (6)

*O + OH^−^ ⇌ *OOH + e^−^        Tafel slope = 40 mV dec^−1^ (7)

*OOH + OH^−^ ⇌ *OO^−^ + H_2_O      Tafel slope = 30 mV dec^−1^ (8)

*OO^−^ ⇌ * + O_2_ + e^−^           Tafel slope = 22 or 40 mV dec^−1^ (9)

The Tafel slope depends on the reaction determining the overall rate (i.e., the rate-determining step), which is a factor relying on the catalyst formulation and the overpotential. Together with each reaction (Equations (1)–(5)), we indicate the Tafel slope for overpotentials between 100 and 400 mV according to the work of Shinagawa and coworkers [41]. An ideal catalyst should provide a catalytic pathway with the rate-determining step being associated with the lowest Tafel slope and towards four electrons for efficient oxygen formation. It is important to point out that a catalyst formulation may present active sites with different natures (presence of defects, different facets, etc.), which can lead to intermediate values of Tafel slopes as a result of the contribution from single active sites to the overall activity. For more details on the theoretical considerations of Tafel slopes in OER, the work of Shinagawa and coworkers is recommended [41].

#### 2.3.1. Metal Hydroxides/Oxyhydroxides

Identification of the active phase in the OER in the case of crystalline metal hydroxides, which is crucial for understanding the reaction mechanism, and a rational design of catalysts are often impeded by the presence of at least two polymorphs, α-phase and β-phase, as in the case of Ni(OH)_2_ and Co(OH)_2_. On the other hand, mixed-metal hydroxide phases have the advantage of a more flexible electron configuration. The addition of a different metallic element to a chosen hydroxide phase allows for the tuning of the electronic structure of the material and hence influences the adsorption energy of the reaction intermediates. Thus, it is one of the most common ways to enhance the reactivity of hydroxide-based materials [42,43]. Oxyhydroxides composed of Ni-Fe and Co-Fe are reported as the most active electrocatalyst for OER in the liquid alkaline electrolytes [44]. It is postulated that the iron dispersed in the matrix of NiOOH or CoOOH forms the actual active sites. Since FeOOH could not serve as a standalone catalytic material due to its electrical insulating properties, the nickel and cobalt oxyhydroxides provide electrical conductivity and chemical stability [45]. Recently, by comparison of Co-rich and Ni-rich (oxy)hydroxide nanosheets, it was found that the increased strength of M–O bonds and porosity at the nanoscale substantially enhance the electrocatalyst stability during the electrochemical reaction [46].

#### 2.3.2. Metal Oxides

Similarly to hydroxide electrocatalysts, first-row transition metal oxides (especially of Ni, Co, and Fe) are the most popular metal oxide materials researched for oxygen evolution reaction in alkaline electrolytes. In particular, spinel- and perovskite-type oxide materials have been extensively studied [47,48,49,50,51,52,53]. For example, in Co_3_O_4_ the generation of oxygen vacancies enhances conductivity, and interactions with the support of or by doping with heteroatoms stabilizes Co^4+^ [54], which leads to the enhancement of electrocatalytic activity [42,43]. In general, stabilization of high oxidation states, as well as Fe^4+^ and Ni^4+^ in oxide matrices (spinel, perovskite), leads to better OER reactivity [42,55,56]. It has to be noted that the structural evolution taking place during electrochemical anodic reaction undergoes a remarkable structural transformation of the metal oxide catalysts under very high potential yields and results in the formation of the oxyhydroxide phase overlayer at the catalyst surface [57]. 

#### 2.3.3. Metal Chalcogenides

Similarly to metal hydroxides and oxides, in the case of transition metal chalcogenides (TMCs) the most promising catalysts are Fe, Co, and Ni-based materials, including metal sulfides, selenides, and tellurides [42,43]. While TMCs have been broadly investigated in the electrocatalytic hydrogen evolution reaction, there is still room for their optimization towards OER.

#### 2.3.4. Metal Nitrides and Phosphides

The high electrical conductivity of metal nitrides renders them ideal candidates for electrocatalytic materials, including oxygen evolution reaction. Similarly to oxide and oxyhydroxide electrocatalysts, mixed metal nitrides (bi- or trimetallic) exhibit a great potential due to the possibility of tuning their electronic properties [42,43]. Although phosphides may exhibit semiconducting or even insulating properties, for a particular atomic ratio of phosphorus and transition metal and suitable electronegativity difference the phosphides may exhibit metallic character, and for some metal-rich compounds superconductivity has been observed [58,59]. Exceptional conductivity of some bi- and trimetallic phosphides is reflected in the similar electronic structure to respective purely metallic materials [60]. As reported in Table 1, FeP/Ni_2_P hybrid electrocatalyst supported 3D Ni foam proved *ex aequo* the best OER reactivity in alkaline conditions. Moreover, it presented very high HER activity, allowing it to act as a bifunctional catalyst for overall water splitting in the same alkaline electrolyte [40]. Thermodynamically, under oxidizing conditions, metal sulfides are less stable than their respective metal oxides, while metal nitrides and phosphides are less stable than sulfides and phosphides, respectively. During strongly oxidative conditions of OER in water, it may be anticipated that metal nitrides and phosphides (and also sulfides, selenides) can easily undergo surface oxidation to the corresponding metal hydroxides or oxides [61,62]. Importantly, oxidation of electrocatalysts, such as cobalt and nickel phosphides in an alkaline environment can be used as an activation process for the oxygen evolution reaction in a neutral solution [63]. 

## 3. Introduction to Carbon-Based Composite Materials

Compared with traditional carbon materials, nanocarbons always exhibit higher electrical conductivity, larger surface area, tunable structural hierarchy, ultra-thin graphitic layer, and low dimensional properties. These unique features endow nanocarbons with multifunctionalities to strongly couple with other catalytically active components, resulting in significantly enhanced performances [64]. In recent years, transition metal/carbon (TM/C) composites have surfaced as a new class of effective catalysts for electrochemical oxygen and hydrogen evolution reactions. These composites usually exhibit the reactivity characteristics of their components (metal compound or carbon); however, the synergetic and interfacial effects are sought for and designed to endorse the material with enhanced electrocatalytic activities. A combination of cheap components, i.e., carbon and Fe-, Co-, Ni-phases and promising reactivities render them perfect candidates for the replacement of precious metal-based electrodes. Thus, new material formulations of the TM/C type, such as hierarchically porous carbon-supported TMs, carbon network supported TMs or carbon encapsulated TMs, are being developed to explore the possibility of deriving new functionalities. Parallel to developing new carbon morphologies, carbon doping, especially with S and N, is used to obtain enhanced properties of the hybrid materials. 

The oxygen evolution reactivities of spinel compounds, which are amongst the most active phases in the OER, are reported to be enhanced by dispersed deposition of spinel oxide phase onto structured carbon substrates such as graphene, single-walled carbon nanotubes (SWCNTs), and carbon nanosheets (CNS). Such a case was reported for the CoCr_2_O_4_, deposited onto carbon nanosheets or Co_3_O_4_ deposited onto carbon porous nanowire array, which displayed excellent OER activities, even outperforming the benchmark RuO_2_ [57]. Another highly active phase in the OER, NiFe hydroxide, was deposited in the form of nanoplates onto different nanocarbon-type materials to improve their electrical conductivity. Various classes of carbon materials were studied, ranging from graphene with different oxidation degrees to amorphous carbons nanoparticles (i.e., Vulcan XC-72R or Ketjen Black) to carbon nanotubes and carbon quantum dots. The composite materials formed with carbon black or carbon nanomaterials usually exhibit higher OER activity than single material type catalysts [65].

The transition metal alloys (TMAs) encapsulated in N-doped graphene are promising hybrid electrocatalysts for water splitting. The enhanced electron transfer at the interface of the TM core and the graphene shell is the main advantage for improved OER reactivity. Moreover, by changing the chemical composition of the transition metal core or thickness of the graphene shell, the electrocatalytic activity may be further tuned to improve the water-splitting activity. Transition metal sulfide (TMS) nanoparticles and nanosheets could be downsized to expose more highly active sulfur edges by a nano-confinement effect of carbon nanomaterials, resulting in the enhanced reactivity of TMSs. Such materials combined into TMS/carbon hybrids can be further enhanced by the rational introduction of another heteroatom, such as nitrogen, or defects into the composite materials. It is anticipated that the TMS/carbon composite materials may be good alternatives to precious metals, even for oxygen evolution reaction in acidic media. In the case of transition metal phosphides (TMPs), TMP/carbon composites are proven to be very effective for water splitting. Similar to other composite materials, the heterointerfaces with carbon are crucial in providing their high OER reactivity. The enhancement of active site exposition and acceleration of mass transport can be obtained in all cases by constructing composite structures based on hierarchically porous carbons, which may allow further improvement of the performance of all TM-phase carbon composites for water splitting [43,47,66,67].

A combination of two types of nanostructured carbon building blocks into nanocomposite structures has also been reported for electrochemical applications in lithium-ion batteries, metal–air batteries as oxygen reduction catalysts and bifunctional catalysts (OER and ORR), and as hydrogen evolving catalysts [67,68,69,70,71,72]. The carbon components are usually 1D structures (e.g., nanotubes) interconnecting 2D or 3D carbons doped with nitrogen to improve their electronic conductivity. In such materials, the carbon/carbon structure acts as a support for well-dispersed, non-noble, metal-based nanoparticles. The enhanced electrocatalytic activity of these materials is ascribed to the optimized surface chemical composition and distribution of the active phase, large specific surface area, and hierarchically porous microstructure resulting in improved electron and mass transport abilities [71,72,73].

The utilization of nanocarbons for high-efficiency hybrid electrocatalysts is based on the following effects: acceleration of the electron and mass transport; modification of the morphologies and properties of active components during the composite/hybrid fabrication; manipulation of the electronic structure through the interfacial charge transfer; generation of confinement effects resulting in (1) spatial restriction to hamper the sintering of nanoparticles, (2) significant electronic interaction to modify the property and activity of carbon shells, and (3) physical isolation of embedded nanoparticles from hostile operating conditions; and supporting electrocatalysts into 3D free-standing electrodes [64].

## 4. Synthesis of Carbon-Based Nanocomposites

In this section, the most popular strategies to prepare carbon-based composites are presented with the details of the synthetic procedures. The preparation of the composites starts with the surface functionalization of carbon components to increase their reactivity. Therefore, the pre-treatment routes of carbons are presented first, followed by a detailed description of the most successful approaches to form composites of carbon with transition metal compounds.

### 4.1. Pre-Treatment of Carbons

**Figure 4:**
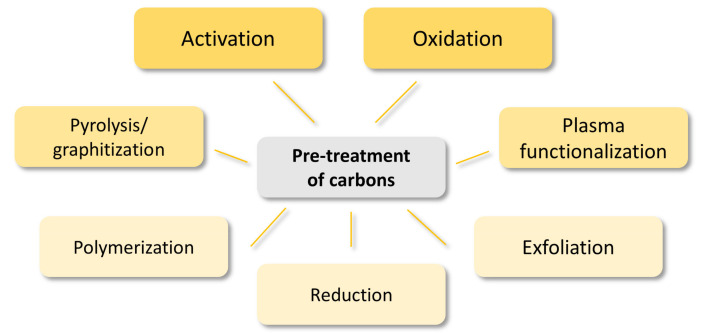
Graphical summary of typical pre-treatment strategies of carbon materials.

Recently, novel synthesis routes for the fabrication of new carbon structures have been extensively explored. The obtained materials can be additionally pre-treated before using as catalysts or supports to enhance their electrochemical performance during the OER process. Pre-treatment of carbons is instrumental in increasing specific surface area, introducing heteroatoms, tuning the electronic structure, improving conductivity, and developing porosity. Herein, common pre-treatment methods of previously fabricated carbon materials, as well as procedures for obtaining hetero-doped structures, will be presented rather than synthesis routes of bare carbons (Figure 4).

- **Pyrolysis/graphitization**

To synthesize a composite consisting of carbon and red phosphorous, the sugar powder and red phosphorous were ground together. The mixture was reacted with a concentrated sulfuric acid to form foam-like carbon. Such obtained material was transformed into a tube furnace and pyrolyzed at 900 °C (3 h) under a flow of argon [74]. As-synthesized ordered mesoporous carbons (SBA-15 silica as a template and a furan resin/acetone mixture as carbon precursor) were subjected to further modifications to enhance the formation of a crystalline carbon framework while maintaining the mesoporous structure. To meet the goal, ordered mesoporous carbons were graphitized at 1500 °C for 1 h under continuous Ar flow [75,76].

- **Polymerization**

The N-doped hollow flower-shaped porous carbon materials (named N-HCNFs) were obtained in a three-step process. Firstly, cyanuric chloride was covalently linked onto mesocrystals of melamine-cyanuric acid (MCA) via a nucleophilic substitution reaction and as a result a covalent triazine framework (CTF) on the MCA surface was formed. Next, by adding piperazine (PP) three complexes with different ratios of CTF:MCA (1:4, 1:2, 1:1) were prepared. In the last stage, CTF-PP@MCA precursors were carbonized at temperatures 800–1000 °C for 2 h under an inert atmosphere of nitrogen [77].

A mesoporous carbon nitride material was synthesized through polymerization of guanidine hydrochloride as a source of carbon–nitrogen inside the organized mesopore channels of a previously prepared mesoporous SBA15 silica template with a 9.15 nm pore size [78]. The latter was impregnated and stirred with the ultrasonicated solution of guanidine hydrochloride for a few hours. The obtained homogenized mixture was firstly heated in an oven at 100 °C h (6 h) and then at 160 °C (6 h). As a result, the dried nanocomposite material was carbonized in a furnace at 500 °C (4 h) under nitrogen flow. To remove the SBA15 silica template, the carbonized sample was stirred in an aqueous solution of hydrogen fluoride. In the last step, a mesoporous carbon nitride material was repeatedly washed with ethanol and dried at 100 °C.

- **Plasma functionalization**

B and N-co-doped carbon catalysts were fabricated through a plasma-assisted synthesis route. The mixtures of boric acid and quinoline with various concentrations (up to 10 mM) were sonicated and plasma discharged. To obtain conductive B,N-co-doped carbon powders, the discharged solution was filtered, dried overnight at 80 °C, and eventually heated to 800 °C in an N_2_ atmosphere for 1 h [79]. Tsounis et al. synthesized vertical graphene (VG) on a carbon fiber paper without using any catalyst in a plasma-enhanced chemical vapor deposition process using Ar, CH_4_, and H_2_ (1000 W, 10 min, 400 °C). To modify pristine vertical graphene, directly after the growth of the material, the plasma chamber was degassed and pumped down to ~10^−3^ Pa. Argon was introduced again to the hot chamber and the VG was post-treated for 5 min. As a result, edge-rich vertical graphene was produced as support for the electrochemically active phase [80]. To form a nanoarray-structurization and N-doping before use as a support for NiFe-P particles, commercial carbon paper was cleaned in ethanol, acetone, and water. Then, the washed pieces of material were dried at room temperature and modified in the N_2_ plasma reactor for 30 min [81].

- **Exfoliation**

Graphene oxide (GO) is usually synthesized from natural flake graphite powder via chemical exfoliation—modified Hummers’ method [82,83]. An acid-assisted liquid exfoliation of bulk graphitic carbon nitride (g-C_3_N_4_) results in nanosheets of carboxyl-functionalized g-C_3_N_4_, which then can be used as part of composite electrocatalysts. In the typical synthesis protocol, powder of bulk g-C_3_N_4_ is mixed with 65 wt.% HNO_3_ and refluxed at 80 °C for 3 h. The precipitates are collected by centrifugation and washed with water. After overnight drying in a vacuum at 60 °C, a powder g-C_3_N_4_ nanosheet is obtained [84]. The exfoliated graphite flakes have been used as a support for iron–nitrogen coordinated carbon nanofiber electrocatalysts. The electrochemical exfoliation of graphite was performed in a two-electrode system using Pt and a graphite flake as counter and working electrodes, respectively, as well as 0.1 M aqueous solution of (NH_4_)_2_SO_4_ as an electrolyte. A direct current voltage was applied to the graphite electrode for 3 min. After the complete process, to remove any residual salts, the exfoliated graphite flake was washed with Milli-Q water. Finally, the received material was dried in the oven [85].

- **Reduction**

Reduced graphene oxide (rGO) was synthesized using previously prepared graphene oxide (GO) via a modified Hummers method [86]. The hydrazine was mixed with a GO aqueous solution at an ultrasound bath. The dispersion was refluxed for 12 h and the resultant material was thoroughly washed with Milli-Q water. Then, the rGO was transferred to an oven and heated at 60 °C for 24 h to obtain a powder. The percentage yield of the graphene oxide reduction was nearly 90%.

- **Oxidation**

Multi-walled carbon nanotubes, MWCNTs, were produced by ethylene chemical vapor deposition in a presence of a Fe-Co catalyst, which then was removed by treatment in boiling HCl solution. The obtained MWCNTs were functionalized with oxygen species through heating under reflux in 68 vol.% nitric acid aqueous solution for 2 h at 120 °C. Such nanotubes were washed, dried and used as a support for metal oxides (M = Mn, Ni, Fe) [87]. Li et al. refluxed pristine carbon nanotubes (CNTs) in a mixture of 65 wt.% HNO_3_ and 98 wt.% H_2_SO_4_ (1:3) at 70 °C for 30 min to remove metal residues and introduced surface oxygen moieties that played the role of anchors for Mn_3_O_4_ adsorption [88]. Similarly, carbon fiber paper has been oxidized in HNO_3_ by heating at reflux. The precipitates were washed with water until they reached pH 7 and then freeze-dried them to obtain the final oxidized powder [89]. Nickel nitride nanoparticles were supported on 2D graphene oxide, which was previously doped with boron. The as-prepared graphene oxide via the modified Hummer method was mixed and ultrasonicated for 2 h with boric acid in deionized water at the following weight ratios H_3_BO_3_:GO = 1:1, 3:1 and 5:1. Afterwards, the mixture was dried at 60 °C and heated at 800 °C for 3 h in an Ar atmosphere [90]. Before the integration of carbon fiber paper (CFP) with a Co-engineered FeOOH catalyst, the commercial carbon material was pretreated. Firstly, CFP was cleaned by ultrasonication in the following liquids: acetone, ethanol, and deionized water. After that, the CFP was modified in concentrated HNO_3_ (65%, aqueous solution) at 115 °C for 3 h in a round-bottom flask equipped with a condenser. The resultant material was washed with water and allowed to dry overnight at room temperature [91].

- **Activation**

Cauliflower leaves were repeatedly washed in Milli-Q water, dried, and were ground into a form of powder. Such carbon precursor was pre-carbonized in a tube furnace (400 °C, 2 h, N_2_ flow). Then, the obtained material was mixed with KOH at the mass ratio of 1:3 and pyrolyzed (800 °C, 1 h, N_2_ flow). To get the biomass-derived activated carbon, the product of pyrolysis was washed with 1 M HCl solution, MilliQ water, and dried overnight (70 °C) [92]. A similar procedure was applied to obtain nanoporous activated carbon sheets that were synthesized from food waste that was high in carbohydrates [93]. Briefly, the food waste material was ground and the resulting powder was heated at 450 °C for 2 h under an inert atmosphere of nitrogen. In the next step, the carbon material was activated with KOH at 750 °C for 3 h (ratio of KOH:C was 1:3). Finally, the obtained activated carbon was washed with water to neutral pH, collected by centrifugation, and dried at 60 °C for 12 h.

The electrochemical technique was proposed to activate carbon cloth, which was used as a support for Co-based films. In a typical procedure, a piece of carbon cloth was immersed in acetone, ethanol, and water, respectively. The activation process was carried out in the two-electrode system in the aqueous solution of 0.1 M (NH_4_)_2_SO_4_ and 2 M NH_4_OH using carbon cloth as the anode and carbon rod as the cathode (a DC voltage of 10 V, 2 min). After the activation, the carbon cloth was thoroughly washed with water [94]. Another activation method of carbon cloth included mild chemical oxidation of raw carbon cloth pieces in an aqueous solution of 98% H_2_SO_4_ and 70% HNO_3_ acids at 70 °C for 24 h. After washing with copious amounts of water and drying, the carbon cloth pieces were transferred to a muffle furnace and calcined at 500 °C for 2 h under an air atmosphere [95].

### 4.2. Synthesis of Carbon–Metal Hydroxides/Oxyhydroxides/Oxides

**Figure 5:**
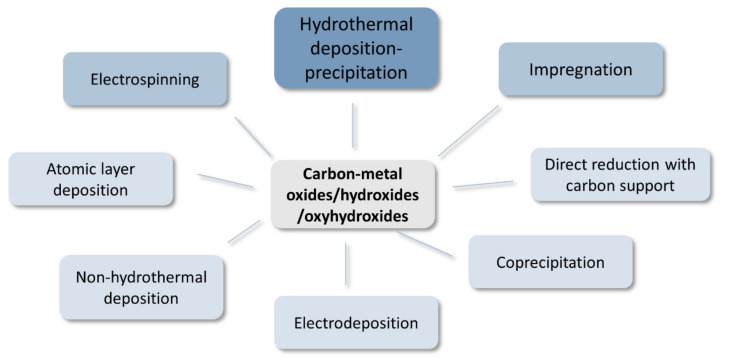
Graphical summary of synthesis methods of carbon–metal hydroxides/oxyhydroxides/oxides.

The great abundance of the hydroxide/oxide carbon materials results from the relative ease of the preparation of the structured catalysts (Figure 5). The synthesis procedures usually do not require high temperature treatments and a variety of precursors can be used. Among the reported synthesis methods, by far the most popular is the hydrothermal treatment of pretreated carbon with metal salt in the Teflon-lined autoclave placed in a conventional oven/dryer.

- **Hydrothermal treatment of the oxide hydroxide precursors with carbon (hydrothermal deposition–precipitation)**

Hydrothermal deposition–precipitation is one of the most popular ways to combine carbons with the hydroxide/oxide phase [96,97]. To combine nitrogen and sulfur co-doped mesoporous carbon spheres obtained by pyrolysis of thiourea with manganese and cobalt spinel oxides, a hydrothermal treatment was implemented. The S,N-doped carbon spheres were added into ultrasonicated anhydrous ethanol to obtain a homogeneous suspension, whereupon metal nitrate solutions were slowly added. After further homogenization of the suspension with stirring, ammonium hydroxide solution was added. The obtained suspension was aged at 85 °C under stirring and further transferred into an autoclave and heated at 180 °C to complete the hydrothermal reaction. The obtained precipitate was recovered by centrifugation, washed with water and ethanol and dried in a vacuum oven at 85 °C [98].

Composite catalysts—Co_3_O_4_ at nitrogen-doped hollow carbon nanospheres were synthesized through a facile, modified hydrothermal process, where desired amounts of presynthesized nitrogen-doped hollow carbon nanospheres and cobalt acetate were added into absolute ethanol and dispersed under ultrasonication, followed by the addition of NH_4_OH aqueous solution under stirring. After stirring at 80 °C, the mixture was loaded into a Teflon-lined stainless-steel autoclave. The hydrothermal process was carried out at 150 °C. After allowing the autoclave to cool down to room temperature, the sample was centrifuged and washed with water and dried at 80 °C overnight. The final product was obtained after annealing the above-resulting powder at 500 °C [99].

To obtain Mn_x_Co_3−x_O_4_ nanocrystals decorated on carbon nanotubes, the CNTs were preoxidized in nitric acid by heating at 110 °C with reflux. Then, they were dispersed in ethanol in a round-bottomed flask and sonicated to ensure uniform dispersion and metal acetates were added. Then ammonia solution was added and the solution was then heated to 80 °C (20 h). Finally, the reaction mixture was transferred to a 100 mL Teflon-lined autoclave for a solvothermal reaction at 150 °C for 3 h. The product was collected by centrifugation, washed with deionized water several times, and freeze-dried to obtain the final product, MCO@NCNTs [89].

Ni/rGO nanoplate composite was prepared via a one-step process with nickel chloride, aluminum chloride, urea, and graphene oxide (GO) suspension as precursors. This mixture was heated to 120 °C and refluxed in nitrogen for 12 h under stirring to obtain a composite of Ni-Al layered double hydroxide (LDH) with GO. The Ni-Al LDH-GO precipitates were repeatedly washed with water and ethanol and then was dried at 60 °C for 8 h in a vacuum. The following reduction reaction was carried in a Teflon-lined autoclave with hydrazine and Ni-Al LDH-GO redispersed in deionized water. After stirring for 30 min, the autoclave was heated to 120 °C for 12 h. The precipitate was washed with deionized water and ethanol a few times and then dried at 60 °C. Then the precipitate was purified by a NaOH solution in another hydrothermal process at 120 °C for 6 h. The black product was washed with water and ethanol and dried [100].

Carbon nanotubes were dispersed in water by continuous ultrasonication. Then, metal nitrates, NH_4_F, and urea were added to the above solution. The obtained homogenate was transferred into a stainless-steel autoclave and heated to 180 °C. After the reaction, the autoclave was allowed to cool naturally. The black products were collected and fully washed with water and anhydrous ethanol. Afterwards, the products were vacuum dried at 80 °C and annealed at 500 °C for 2 h under an Ar atmosphere. Next, the products were annealed at 500 °C for 1 h under an Ar/NH_3_ mixed atmosphere and cooled under an Ar atmosphere to obtain final products [101].

- **Non-hydrothermal deposition**

In the deposition–precipitation process, a NCNT/CNT forest was immersed in a solution of precursors containing cobalt acetate, KOH, and H_2_O_2_ for 30 min for sufficient infiltration of the material by the reactants. Then, the temperature of the precursor solution was elevated to 45 °C and maintained for 90 min. The resultant cobalt hydroxide coated NCNT/CNT forest was moved to deionized water for rinsing. The residual water trapped in the hybrid catalyst forest was removed by rapid vacuum drying to maintain the vertical forest morphology. As prepared amorphous cobalt hydroxide coated NCNT forest was 400 °C rapid heat-treated to get amorphous cobalt oxide coated NCNTs, and 700 °C rapid heat-treated to get crystalline cobalt oxide anchored NCNTs (CoO-NCNTs) [102].

In the direct combination of metal oxide with carbon material, Mn_3_O_4_-rGO-x was obtained by coating GO nanosheets on hydroxyl-modified multishell Mn_3_O_4_ hollow spheres and the subsequent reduction of graphene oxide through thermal treatment. The multishell Mn_3_O_4_ hollow spheres were dispersed in water containing ammonium hydroxide to obtain hydroxyl-modified multishelled Mn_3_O_4_ hollow spheres. The washed product was redispersed in water and combined with the suspension of GO and stirred. The obtained solid was recovered by centrifugation and annealed at 400 °C in an Ar atmosphere to obtain rGO. This procedure utilizes the affinity of the OH groups for the surface oxygen vacancy sites on the Mn_3_O_4_ phase. The oxygen functional groups on the GO surface act as the oxidation sites, enabling strong bonding between manganese spinel and graphene, which results in a uniform dispersion of multishell Mn_3_O_4_ spheres encapsulated in graphene. This specific interaction of manganese with carbon through the oxygen atom (Mn-O-C) combined with abundant oxygen vacancies on the Mn_3_O_4_ surface results in a coupling effect, which produces a high density of active sites and improved electron transfer properties [103].

- **Electrospinning of precursors**

Electrospun NiMn_2_O_4_ and NiCo_2_O_4_ spinel oxides supported on carbon nanofibers from metal acetates and polyacrylonitrile (PAN) as metal oxides and carbon nanofiber precursors in dimethylformamide (DMF). The electrospun layer was subjected to an initial stabilization thermal treatment at 270 °C in air and subsequent carbonization at 900 °C under helium flow. Finally, an oxidation thermal treatment was carried out at 350 °C in static air to obtain the composite materials [104].

PVP and metal nitrates are added to DMF and stirred at ambient conditions. Afterwards, the homogeneous solution was filled into a plastic syringe equipped with a 22-gauge needle tip and a DC voltage of 20 kV was applied to the electrospinning set up. The as-obtained nanofiber membrane was initially stabilized at 250 °C for 3 h in the open air, and subsequently by annealing at 600 °C for carbonization for 3 h in N_2_ atmosphere to synthesize the final hollow-Co_3_O_4_/CeO_2_@N-CNFs [105].

- **Impregnation**

To obtain a mixed transition metal (Fe, Co, Ni) oxide, nanoparticles supported on oxidized multi-walled carbon nanotubes, MWCNT were mildly oxidized in concentrated HNO_3_, washed with water, and dried in air to obtain oxygen functionalized nanotubes. Preparation of Fe, Co, Ni, and mixed Fe–Ni–Co oxide/MWCNT catalysts was performed by incipient wet impregnation of oxygen functionalized MWCNT with aqueous solutions of metal nitrates. The impregnated samples were dried at 110 °C and subsequently annealed at 350 °C under argon flow. The concentration of Fe, Co, and Ni nitrates in the impregnation solutions was varied to obtain composite samples containing different metal ratios while keeping the total metal loading at about 14 wt.% [106].

For a typical synthesis of Ni/NiO/N-doped active carbon hybrids, the as-prepared active carbon and nickel nitrate were added into ethanol, and after ultrasonic treatment, the mixture was kept to 80 °C under vigorous stirring to evaporate the ethanol. The resultant solid was transferred into a tube furnace in an N_2_ environment and annealed at 400–600 °C for 2 h to obtain the final product [92].

- **Direct reduction with carbon support**

Reduced carbon layers, differing in lateral dimension, were used as a reduction agent for iron(II) salts, resulting in the decoration of the carbon framework by iron oxide nanoparticles. Depending on the choice of the carbon starting material, the average size of the formed nanoparticles was situated between 3–50 nm. To obtain the composite material, graphenide solutions from different graphite starting materials have been used directly as a reduction agent for iron chloride dissolved in absolute tetrahydrofuran (THF) inside an argon-filled glove box. After the aggregation and precipitation occurred, the respective dispersion was further stirred, and afterwards, the dispersion was centrifuged in the glove box and the supernatant was removed. The samples have been redispersed in THF followed by another centrifugation step. Afterwards, the samples were removed from the glove box and water was added. The sample has been mixed with cyclohexane and has been extracted three times with water in a separation funnel. The final material was collected via filtration (0.2 µm filter membranes) and dried in a vacuum [107].

- **Electrodeposition**

Mixed Ni–Fe hydroxides were electrochemically deposited on graphene, Ni-foam, Cu-foam, and graphene paper in an aqueous-based solution with metal nitrate. Potentiostatic electrodeposition was sustained for 100 s with an applied potential of 1.1 V vs. Ag/AgCl using a graphite rod counter electrode. After electrodeposition, the samples were washed thoroughly in water and ethanol [80].

- **Coprecipitation**

The ternary NiFeMn-Prussian blue analogues/polyvinylpyrrolidone (NiFeMn-PBA/PVP) hybrid precursors were prepared by a modified coprecipitation method. Metal acetates and PVP were dissolved in alcohol. K_3_[Fe(CN)_6_] was dissolved in water to form another solution, which was added dropwise into the solution with acetates and PVP under intense stirring at room temperature. The resulting aqueous solution was aged for 2 h, and the precipitate was obtained by centrifugation without washing. Afterwards, the precipitate was dried at 80 °C. The above was calcined under Ar atmosphere at 700 °C [108].

- **Atomic layer deposition**

Bis(1,4-diiso-propyl-1,4-diazabuta-diene) cobalt precursor (Co(dpdab)_2_) was used for the deposition of CoO_x_. Ozone was selected as the oxidant for the atomic layer deposition (ALD) reaction. Before deposition, carbon paper (CP) was ultrasonically cleaned with acetone, methanol, and deionized water. The chamber pressure was maintained at 0.1 Torr using a rotary pump. The chamber and cobalt precursor were heated to 200 and 112.5 °C, respectively. During the deposition, high-purity nitrogen gas was allowed to flow through the chamber to purge by-products and excess chemicals. One cycle of ALD-CoO_x_ on CP consisted of N_2_ pulse/Co(dpdab)_2_ pulse/N_2_ purge/O_3_ pulse. The growth rate of ALD-CoO_x_ was 0.4 A/cycle. The deposition of ALD-CoO_x_ was carried out with different numbers of cycles (50, 100, 350, and 700) [109].

### 4.3. Synthesis of Carbon–Metal Chalcogenides

**Figure 6:**
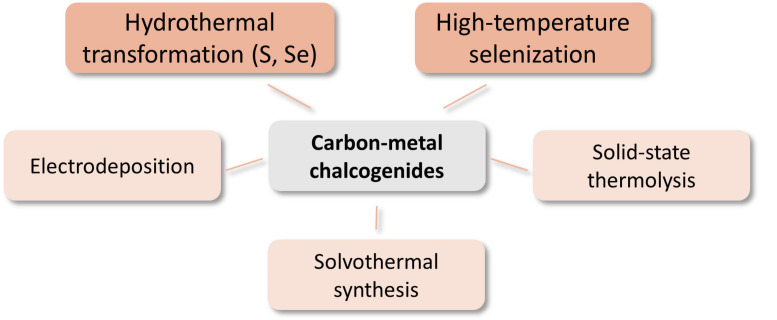
Graphical summary of synthesis methods of carbon–metal chalcogenides.

Although from a thermodynamical point of view, metal sulfides are less stable than metal oxides under oxidizing potentials and metal nitrides and phosphides are less stable than sulfides and so on, an abundance of such electrocatalytic systems has been reported in recent years also for the OER. It can thus be anticipated that metal chalcogenides, nitrides, and phosphides would be readily oxidized, at least on the surface, to the respective metal oxides/hydroxides in the strongly oxidative environments of OER [61]. Therefore, care must be taken when characterizing such systems and particular attention should be paid to the spent catalysts. Most popular preparation methods are summarized in Figure 6.

- **Hydrothermal transformation**

Hydrothermal treatment is frequently used to convert metal precursors to respective metal sulfides or selenides. Usually, pre-oxidized carbon materials and metal nitrates, acetates, or chlorides and chalcogenide sources are used in a Teflon-lined autoclave to carry out the reaction at temperatures from 120 to 200 °C for 2–20 h. Initially, the metal precursor hydrolyses on the surface of oxidized carbon support and then is converted into metal sulfide. Selenides can be obtained with the use of selenide powder and an additional reducing agent.

The Co@NC@MoS_2_ catalyst was synthesized using a hydrothermal treatment of the Co@NC nanoparticles, where Co@NC nanoparticles were dispersed in deionized water and sonicated for 1 h. Afterwards (NH_4_)_6_-Mo_7_O_24_·4H_2_O and L-cysteine were added to the Co@NC dispersion and again sonicated for 1 h. The obtained dispersion was transferred into a Teflon-lined autoclave and heated at 200 °C for 20 h. The cooled-down precipitate was recovered by centrifugation, washed with ethanol and water, and dried in a vacuum dryer [110]. A Co_9_S_8_/graphene composite was obtained with sodium sulfide (Na_2_S) solution mixed with a graphene dispersion followed by ultrasonication and transferred to an autoclave where subsequently cobalt nitrate solution was poured in with gentle stirring whereupon a black gel was formed. The hydrothermal reaction was carried out at 120 °C for 3 h, the precipitate was washed with water and ethanol, and the product was collected after lyophilisation [111]. In a synthesis of NiCo_2_S_4_/N-CNT, mildly oxidized CNTs were ultrasonically dispersed in ethanol and deionized water mixture. Then, stoichiometric amounts of Co(AC)_2_·4H_2_O and Ni(AC)_2_·4H_2_O were dissolved in this suspension and stirred at 80 °C for 2 h in a water bath, followed by the addition of NH_3_·H_2_O. After further stirring, thiourea was added and after continuous stirring for 20 h, the reaction mixture was transferred to an autoclave for solvothermal reaction at 170 °C for 3 h. The precipitate was washed with water and ethanol and the product was collected after lyophilisation [112].

Carbon nitride-nickel selenide material was prepared from the graphitic C_3_N_4_, nickel chloride and Se powder as precursors in an aqueous solution with NaBH_4_ as a reducing agent. Se/NaBH_4_ solution and g-C_3_N_4_/Ni^2+^ suspension were mixed, stirred for 1 h, and transferred into a Teflon-lined autoclave for hydrothermal treatment at 160 °C for 12 h. The resulting product was collected by filtration, washed with deionized water and ethanol, and then dried at 60 °C overnight [113]. Ni/Co/CoO/NiCo_2_O_4_–g-C_3_N_4_ prepared by thermal decomposition was used as a precursor for a CoSe_2_/Ni_3_Se_4_@N-doped carbon nanosheets/ketjen black carbon composite electrocatalyst. The precursor, carbon support, and SeO_2_ were dispersed in dimethylformamide aqueous solution and were transferred into a Teflon-lined stainless steel autoclave and kept at 200 °C for 20 h. The obtained precipitate was collected by centrifugation, washed with water, and dried in a vacuum at 80 °C. However, to obtain complete selenization, an additional pyrolysis step at 700 °C in an inert atmosphere was needed [114]. A similar two-step procedure, with thiourea as S precursor and nitrates as metal precursors and an additional pyrolysis step at 900 °C, was used to obtain NiCo_2_S_4_/carbon-nitrogen nanosheet composites [84].

Other selenium precursors for autoclave selenization can be used, such as Se powder dissolved in a solution of ethylenediamine and ethylene glycol [115] or diphenyl diselenide dispersed in ethanol [116]. The latter was also used in combination with sulfourea to obtain Co(S_x_Se_1−x_)_2_@C materials.

- **High temperature selenization**

This method is most frequently applied for direct selenization of the metal precursor and is usually carried out in a tubular furnace where Se powder (but also S or Te) is placed in a crucible in the upstream side of the tube and the metal/carbon precursor downstream. The furnace is purged with an inert gas, usually argon, and kept between 350 and 500 °C (usually 450 °C) for 2–3 h. The precursor is frequently in the form of a Prussian blue analogue (PBA) [117,118] or a zeolitic imidazolate framework (ZIF) type metal–organic framework (MOF) compound where the metal is reduced before selenization [119,120].

In one typical synthetic procedure, the as-prepared PBA metal precursor deposited onto the carbon support and Se powder were put at two separate positions of a quartz boat with Se powder at the upstream side of the tube furnace. Then, the samples were annealed at 350 °C for 2 h in N_2_ atmosphere with a heating rate of 2 °C min^−1^ to obtain Ni–Fe–Se/N-CNTs [117]. Another bimetallic NiFe PBAs as MOF precursors were electrodeposited on carbon fiber paper (CFP) and were put inside a tube furnace with the Se powder in a typical arrangement. The temperature was increased up to 450 °C at a ramping rate of 5 °C min^−1^ and kept there for 30 min in Ar atmosphere to obtain NiFe-Se/CFP [118]. Synthesis protocols involving ZIF compounds as metal precursors follow a similar scheme. A decomposed and reduced MOF material, previously deposited onto a N co-doped carbon nanopolyhedra/nanotubes support, Co@NC-CNTs, and the Se powder were placed in quartz boats in a vacuum tube furnace with flowing nitrogen and annealed at 500 °C for 3 h with a heating rate of 5 °C min^−1^ [119]. Carbon nanotubes grafted 3D core–shell CoSe_2_@C composites were obtained by grinding together the synthesized ZIF material and commercial selenium powder and heating under Ar flow to 450 °C with 5 °C min^−1^ heating rate and annealing for 2 h [120]. ZIF precursor can also be used to obtain a sulfide electrocatalyst by using a three-stage heating procedure. To obtain the CoS deposited on carbon fiber (CF), a piece of cotton cloth with a deposited ZIF-L was put in the high temperature area of a tube furnace, and the excess S powder was placed in the upstream of a quartz tube in a porcelain crucible. With argon flowing, the temperature was raised to 350 °C at a heating rate of 2 °C min^−1^ and maintained for 30 min. Then, still with argon, the temperature was further raised to 800 °C at a heating rate of 5 °C min^−1^ and kept for 90 min. Next, the Ar flushed furnace was cooled down to 200 °C at a rate of 5 °C min^−1^ and held for 2 h to remove residual S powder [121]. Also, a different type of MOF, such as MOF-74, can be used as both metal and carbon precursor to obtain a CoSe_2_/FeSe_2_ carbon nanorods electrocatalyst. As-synthesized MOF-74-Co/Fe precursor was fully mixed with commercial selenium powder in the mass ratio of 1:2 by grinding and then was transferred into a quartz boat. The temperature of the furnace was risen to 450 °C with a heating rate of 2 °C min^−1^ under an argon atmosphere and maintained for 2 h [122].

- **Other methods**

Other methods for chalcogenide-carbon composite synthesis include solid-state thermolysis, solvothermal synthesis, electrodeposition, and vulcanization. In the **solid-state thermolysis method**, carbon and chalcogenide components are obtained simultaneously during the high temperature treatment of precursors. In a simple synthesis, cobalt nitrate as a metal precursor and thioamide as sulfur precursor mixed uniformly can be pyrolyzed at 900 °C under Ar flow to yield Co/Co_9_S_8_/CNT [123]. Similarly, a metal precursor, such as Co-based Prussian blue analogue, can be ground together with the elemental chalcogenide (S, Se, Te) powder and placed inside a closed stainless steel tube. The reaction is carried out for 3 h at 500 °C and the products comprised of only one phase chalcogenide product [124]. **Solvothermal synthesis** with non-aqueous solvent is carried out in a typical Teflon-lined autoclave reactor where the carbon support is previously covered with metal compounds, e.g., by electrochemical deposition and with an addition of a reductor, e.g., NaBH_4_ and usually Se powder. Care is taken to remove the dissolved oxygen by purging the solution with inert gas before sealing it in the autoclave. In this type of synthesis, ethanol was used as a solvent to obtain NiSe/CFs [125], and N, N-dimethylformamide to obtain trimetallic NiFeCoSe_x_/CF cloth materials [126]. In the **electrodeposition** method, the chalcogenide compound on carbon support is directly obtained during the electrochemical reaction. The chronoamperometric method was used to obtain cobalt-sulfide sheets on carbon paper/carbon tubes material (CP/CT). A CP/CT electrode was subjected to a constant potential of −0.9 V vs. Ag/AgCl_2_ in a cobalt nitrate, thiourea in ethanol-water solution for 8 min. After drying and annealing under Ar atmosphere at 300 °C for 1 h, the Co-S sheets on the CP/CTs were obtained [127].

### 4.4. Synthesis of Carbon-Nitrides and -Phosphides

**Figure 7:**
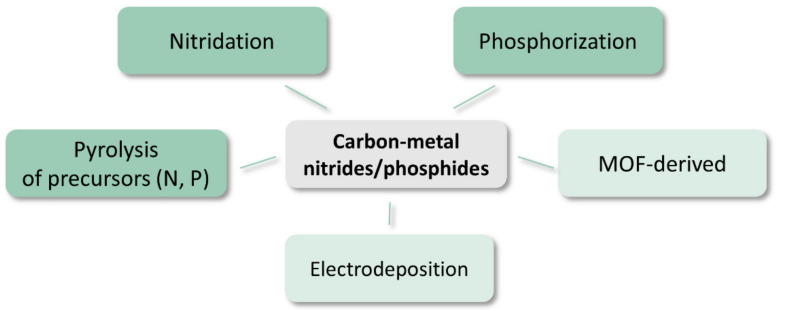
Graphical summary of synthesis methods of carbon–metal nitrides and phosphides.

Recent reviews describing the properties and electrochemical applications of metal phosphides are presented in [60,128], while metal nitrides are reviewed in [129,130]. Carbon composite materials can be obtained by a procedure involving a direct combination of components, e.g., ultrasonication of Ni_12_P_5_ and oxidized MWCNTs can be used [131]. However, most of the described methods involve the preparation of a precursor that is then high temperature treated to obtain the final material (Figure 7).

- **Pyrolysis of precursors**

Pyrolysis can be used to obtain both carbon-nitride and carbon-phosphide composite materials. Using a general strategy combining sol–gel and carbonization-assisted route phosphides of Co, Mo, Fe, Ni, and their combinations can be coupled with an amorphous carbon matrix in a one-step carbon composite formation. In this procedure, metal chlorides, NH_4_H_2_PO_4_, citric acid, and deionized water were mixed to form a homogeneous aqueous solution, which was refluxed at 80 °C for 20 min to form a sol. Then, the sol was dried at 140 °C to obtain gel ash. The gel ash was collected and heated at 900 °C in an H_2_/Ar atmosphere to obtain carbon–metal phosphide nanoparticle materials [132]. Another recently reported method to prepare supported transition-metal phosphide catalysts is the so-called carbothermal hydrogen route (carbothermal hydrogen reduction method). Cobalt nitrate and carbon black powder with ultrapure water were mixed with a solution of the phosphorus precursor, (NH_4_)_2_HPO_4_, and sonicated to form a well-dispersed metal phosphate (M_x_(PO_4_)_y_) on carbon black. Then, the sample was dried, ground and pyrolyzed in H_2_/N_2_ atmosphere at 1000 °C to obtain 40 wt.% Co_2_P on carbon black [133]. Phosphide and nitride carbon composites can also be prepared by electrospinning and subsequent pyrolysis, such as in the procedure where DNA, PAN, and cobalt acetate were used as substrates for electrospinning. The electrospun precursor was then dried in air at 270 °C and pyrolyzed at 900 °C in N_2_ atmosphere to derive a Co_2_P/Co_2_N core–shell nanostructure embedded in N-doped carbon nanofiber [134].

- **Nitridation of metal precursors deposited on the carbon matrix**

Metal salts can be hydrolyzed on the surface of the pre-treated carbon materials with subsequent conditioning in hydrothermal conditions. In the case of nitride materials, the nitridation in the NH_3_ atmosphere at elevated temperatures completes the preparation procedure. Usually, carbon matrix, metal salt (acetate, nitrate), and hydrolyzing agent (hexamethylenetetramine, dimethylamine) are put into Teflon-lined autoclaves and heated at about 120–160 °C for several hours (e.g., 6–12 h). The obtained hydroxides/carbon hybrids are further annealed under ammonia flow, usually at 400–500 °C for 2 h [135,136]. An alternative way of preparing a precursor for nitridation may involve heating the carbon matrix and metal salt (e.g., chloride) with the precipitating agent in the form of trisodium citrate in an oil bath at 90 °C [137]. The following nitridation is the same as above. If the carbon material already contains metal, such as Ni, the addition of iron salt results in a galvanic replacement mediated method to obtain iron–nickel nitride active phase on a carbon support [137]. Tannic acid can also be used to precipitate precursors onto carbon support at room temperature [138]. In this case, nitridation was carried out at 750 °C with an N_2_:NH_3_ ratio of 3:1 for 1 h.

- **Phosphorization of metal precursors with NaH_2_PO_2_**

Similar to precursors for nitridation, the hydrothermal process can be used to obtain hydroxides for subsequent phosphorization. Usually, a carbon matrix, metal salt, and a hydrolyzing agent (hexamethylenetetramine, urea) are put into Teflon-lined autoclaves and heated at about 120 °C for several hours (i.e., 6–12 h). Afterwards, the precursor and excess of NaH_2_PO_2_ are put into a quartz boat at different positions with the latter at the upstream side and heated at 300–350 °C for 2 h in an inert atmosphere [139,140,141]. Deposition in a chronoamperometry mode can also be used to prepare precursors for phosphorization, as in the deposition of polyaniline on carbon cloth followed by deposition of Ni at −10 mA cm^−2^. In this case, however, the final phosphide electrocatalyst required additional annealing at 800 °C after the typical phosphorization procedure [142].

- **Electrodeposition of phosphide phase**

This method stands out among the reported procedures because it can be a one-step synthesis. The electrodeposition process can be used to deposit amorphous phosphide precursors on carbon electrodes, which may or may not be further annealed. Usually, a solution with metal salt (nitrate, chloride), trisodium citrate, and NaH_2_PO_2_ is used in a three-electrode system. Cyclic voltammetry (20 cycles at 5 mV s^−1^ from −1.3 to −0.3 V vs. Ag/AgCl) or chronopotentiometry (−1.2 V vs. saturated calomel electrode) modes can be used for deposition of metal phosphide phase [81,143].

- **Metal–organic framework-derived**

Zeolitic imidazolate framework (ZIF) materials are often used to obtain carbon–metal composites, especially combined with carbon cloth. Usually, Co-ZIF material is synthesized in the presence of carbon cloth with a reaction time of up to 5 h. Afterwards, the precursor may be directly subjected to a nitridation process at 400 °C [144], or firstly decomposed at 800 °C in a reducing atmosphere and then nitride [145]. A phosphide electrocatalyst can be achieved by simultaneous annealing and phosphating of a metal-rich metal–organic frameworks precursor. The as-synthesized MOF and NaH_2_PO_2_ are used as in the typical phosphorization procedure but at a higher annealing temperature of 650 °C [146].

- **Phosphides and nitrides with protective carbon shell**

A protective carbon layer over metal phosphides is used to prevent the leaching of metal ions during oxidative reactions. A simple procedure involves covering the phosphide material with glucose in a Teflon-lined autoclave in a hydrothermal process with subsequent carbonization at 400 °C for 2 h in an inert atmosphere [147]. Alternatively, a precursor in form of 1,2-bis-diphenylphosphinoethane dichloronickel (II) can be pyrolyzed in a reducing atmosphere at 400 to 600 °C for 2 h to obtain carbon-encapsulated nickel phosphide nanoparticles [148]. For nitride materials with carbon shell, Co_3_[Co(CN)_6_]_2_—Prussian blue analogue precursors were subjected to calcination and nitridation in the N_2_/NH_3_ atmosphere at temperatures of 450–650 °C for 2 h to synthesize cobalt nitrides, Co_3_N, Co_3_N/Co_4_N, and Co_4_N [149].

## 5. Reactivity of Carbon-Based Composite Materials

In this review, the carbon-based electrocatalysts are classified according to their composition into four categories: (i) metal-free carbon composites (ii) composites of carbon with metal hydroxides/oxyhydroxides/oxides, (iii) composites of carbon with metal nitrides/phosphides, and (iv) carbon–metal chalcogenides. Density functional theory (DFT) calculations are frequently used to rationalize the observed reactivity trends, and such results are also often presented here. The full review of the theoretical methods, however, is beyond the scope of the present article.

**Figure 8:**
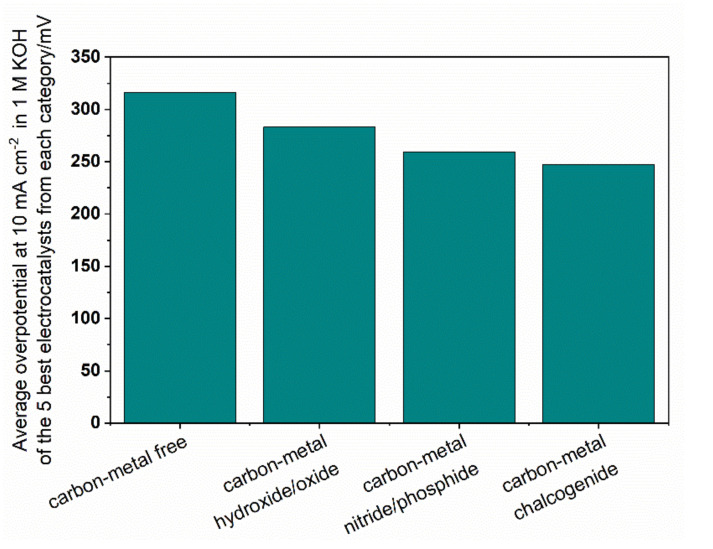
Average overpotentials at 10 mA cm^−2^ of the five best carbon-based electrocatalysts reviewed from each category in 1 M KOH.

From the point of view of the carbon component, the highest reported activities in OER are, in general, obtained for the composites with graphene or reduced graphene oxide [80,100], and N or S,N-co-doped graphene materials [96,150,151,152]. Interestingly, similarly high reactivities can be obtained with composites built with amorphous carbons without [147,153], or with alien atom doping [139,154]. Heteroatom doping of carbon materials results in improved oxygen evolution activity. A straightforward comparison of the classes of the electrocatalysts is difficult and ambiguous due to the limited database of explored materials. Nonetheless, the average overpotential for the 5 best electrocatalysts studied in 1 M electrolyte, in each class reported in this review, may be a useful first approximation to evaluate the carbon-based composites in OER (Figure 8). The average overpotential values are: (i) 316, (ii) 283, (iii) 259, and (iv) 247 mV at 10 mA cm^−2^, for metal-free carbons, oxides, nitrides/phosphides and chalcogenides, respectively. Thus, in terms of a generalized trend in OER reactivity, the most active catalysts are found in the chalcogenide category, followed by the metal nitrides/phosphides. The least active composites are formed without the addition of metal compounds. In the following sections, the most active OER electrocatalysts are presented in detail, with discussions on the possible origins of the observed reactivity.

### 5.1. Metal-Free Carbon Composite Electrocatalysts

**Figure 9:**
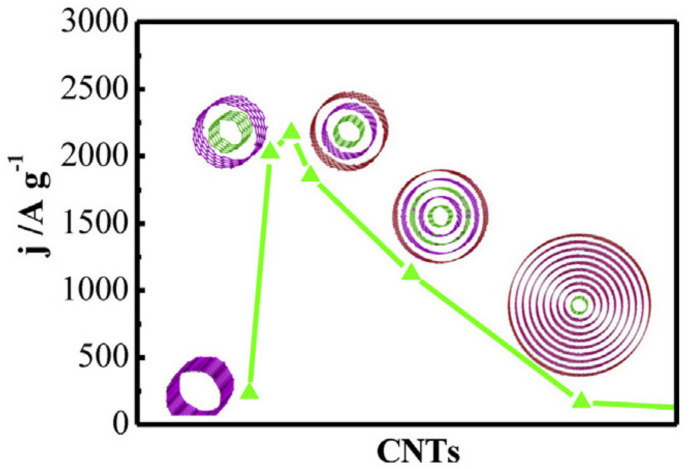
A plot of the activity of CNTs for the OER in 1 M KOH solutions as a function of the number of walls. The mass-specific activity was measured at 1.8 V (vs. RHE) at a scan rate of 1 mV s^−1^ and rotating rate of 2000 rpm with CNTs loading of 0.025 mg cm^−2^. Reprinted from Applied Catalysis B: Environmental, 163, Yi Cheng, Changwei Xu, Lichao Jia, Julian D. Gale, Lili Zhang, Chang Liu, Pei Kang Shen, San Ping Jiang, Pristine carbon nanotubes as non-metal electrocatalysts for oxygen evolution reaction of water splitting, 96–104. Copyright (2015), with permission from Elsevier [155].

One of the ongoing issues concerning the production of the highly active and stable catalysts for water splitting is the cost of the metal active phase. Among metal-free alternatives, carbon-based electrodes have recently attracted great attention due to their relatively low price and robustness (mechanical strength, electrical conductivity) which is of paramount importance while designing OER electrocatalysts for large-scale application. A broad family of carbon-based nanomaterials can be fabricated offering various structures and dimensionalities: 0D (fullerenes, carbon dots), 1D (nanotubes, nanofibers), 2D (graphene and graphemic materials, such as few-layer graphenes, graphene nanoribbons), or 3D (capsules, spheres, nanoporous structures). Such structural diversity has an immediate impact on the physicochemical properties of the materials and hence OER activity. The characteristic reactivity parameters of the metal-free carbon electrocatalysts are summarized in Table 2. Pristine carbon-based materials, mostly carbon nanotubes (CNTs), have recently been investigated as OER catalysts. Cheng et al. revealed that pristine CNTs with specific characteristics, namely an outer diameter of 2–5 nm and the number of concentric tubes of 2–7, exhibit an excellent electrocatalytic activity toward OER in alkaline medium, which follows the volcano-type dependence on the number of walls of CNTs (Figure 9) [155].

The current density of triple-walled nanotubes is ~56 mA cm^−2^ at 1.8 V in 1 M KOH, whereas the values measured for SWCNTs and MWCNTs are around 10 and 35 times smaller, respectively. An excellent activity of nanotubes with the specific number of walls at high polarization potentials, much better even than for 50% Pt/C (5.6 mA cm^−2^), can be explained by the reactive sites on the outer wall for adsorption/dissociation of -OH^−^ and -OOH* species and by the electron tunnelling between the outer wall and inner tubes. As a result, OER, which is a charge transfer dependent process, is promoted at the surface of the outer wall of the carbon nanotubes. Such improvement of the activity is impossible for SWCNTs, whereas reduced driving force across the walls of MWCNT leads to decreased effective electron tunnelling between the outer wall and inner tubes. Moreover, Cheng et al. conducted experiments that excluded the importance of trace metal impurities in differences of the activities between nanotubes with various numbers of walls. A similar conclusion was drawn by Ali et al. [161], who observed that the onset potential of CNTs (four concentric tubes without any post-treatment) for the OER process in 3 M NaOH is reduced (1.60 V) compared to a Pt-based electrode (1.72 V). The overpotential of materials decreases with an increased concentration of the electrolyte.

The susceptibility of carbon materials to chemical reactions is linked to topological and edge defects. Nevertheless, to boost intrinsic OER activity, pristine materials usually require surface and/or structural modifications that at present are often realized by the introduction of heteroatoms, such as O, N, S, F, B, and the formation of stable surface-specific functional groups. Furthermore, doping leads to changes in the charge distribution and electronic properties as well as the formation of additional defects, besides the intrinsic ones, enhancing the electrocatalytic performance of carbons. The extent of possible modifications depends on the electronegativity and size of the doping atoms. 

An important aspect of doped catalysts is their stability in harsh reaction conditions (high OER potential and corrosive environment), since the oxidation of carbon, or heteroatom, is thermodynamically favorable. From a practical point of view, stability is even more important than the initial reactivity of the novel heteroatom-doped carbon material. Finally, the purity of the heteroatom-doped concerning trace metals should be carefully evaluated. The functionalization procedures may lead to the unintentional introduction of traces of metals that may be responsible for the significant improvement of the electrocatalytic performance. 

Yang et al. reported that nitrogen-doped 3D graphene nanoribbon exhibited excellent OER activity with an overpotential of 360 mV at 10 mA cm^−2^ in 1 M KOH and Tafel slope of 47 mV dec^−1^. Authors experimentally proved thorough X-ray absorption near-edge structure (XANES) spectroscopic measurements that the electron-withdrawing pyridinic N groups play a role as active sites for OER [156]. Such groups provide p-type doping effects, accepting electrons from adjacent C atoms and facilitating the adsorption of OH^−^/OOH^−^ water oxidation intermediates. Davodi et al. demonstrated excellent activity of N-doped MWCNTs towards both OER and HER. The electrode required an overpotential of 360 mV and 340 mV toward OER and HER respectively, to achieve 10 mA cm^−2^ in 0.1 M NaOH [158]. Jiang et al. presented nitrogen-doped ultrathin carbon sheets with a high surface area (1793 m^2^ g^−1^) and rich edge defects. Such materials can be used as a trifunctional electrode for OER, ORR, and HER with high performance and durability. DFT calculations revealed that the carbon atoms located at the armchair edge and adjacent to the graphitic N dopants act as the active site for the electrocatalytic reactions [162]. N-doped cotton cloth requires a low overpotential of 351 mV to deliver 10 mA cm^−2^ of current density with a Tafel slope of 88 mV dec^−1^ during the OER test, while the parental material exhibited 382 mV and Tafel was 118 mV dec^−1^ [157]. Another approach for the improvement of catalytic activity toward OER and catalysts stability consists in surface oxidation of the raw carbons. Introduction of oxygen-containing functional groups into carbon cloth surface (O/C ratio of 0.051) allowed the reaching of an overpotential of 477 mV, giving a current density of 10 mA cm^−2^ with a Tafel slope of 82 mV dec^−1^ and long-term durability (at least 24,000 s) during OER tests in 0.1 M KOH [160]. Under alkaline conditions, ketonic -C=O sites make substantial contributions to the OER activity of carbon-based electrodes since by altering the electronic distribution of the surrounding carbon atoms they facilitate the electron transfer and adsorption of water oxidation intermediates [160,163].

Furthermore, the OER activity of the N-doped carbons can be enhanced by the synergistic effect of the introduced multiple heteroatoms. Incorporation of boron was reported to decrease the onset potential by 100 mV vs. RHE and overpotential by 61 mV at current densities of 10 mA cm^−2^ in alkaline media compared with the corresponding N-doped catalyst. DFT verified that chemisorption energy of -OH* on B,N-co-doped catalyst was 0.281 eV less [79]. Similarly, Paul et al. documented an improved performance of graphene/BN-graphene stacked nanofilms toward OER due to the modified π-bonding environment in the top graphenic layer by doped B and N atoms. As a result, the electron transfer during OER tests was facilitated. The material reached an onset potential of 1.6 V vs. RHE and Tafel slope of 143.22 mV dec^−1^ [159]. Li et al. fabricated N and P dual-doped graphene/carbon nanosheet (N,P-GCNS) composites that exhibited an OER onset potential of 1.32 V, whereas for RuO_2_, which is one of the most popular OER electrocatalysts, the value was 1.39 V. The current densities at 1.9 V were 70.75 and 32.41 mA cm^−2^ for N,P-GCNS and RuO_2_, respectively [164].

Besides co-doping, one important strategy to enhance the electrocatalytic kinetics is microstructure engineering of the non-metal carbonaceous catalysts. In the case of 2D graphenic-based catalysts, the electron transfer can be diminished due to the susceptibility of these materials to stacking together that results in shielding of the active sites. Thus, 3D carbon materials, such as carbon hydrogels, foams, and hierarchical porous carbons are commonly used as electrode materials or supports for energy storage and conversion devices [165,166,167]. The porous structure allows for the increase in the utilization efficiency of the catalytically active centers, providing access to the sites to the electrolyte and reactants. Guo et al. revealed that immobilization of the nitrogen and sulfur co-doped graphene flakes on the interconnected conductive graphite foam facilities electron transfer and decreases electrolyte/electrode interface resistance during the OER process [151]. Such composite material can act as a free-standing electrode that exhibits an initial overpotential of 330 mV vs. RHE at 10 mA cm^−2^ in 1 M KOH with a Tafel slope of 149 mV dec^−1^. The synergistic effect of nitrogen and sulfur as co-dopants, as well as the importance of the 3D nanoarchitecture to provide a high density of the OER active sites and promote the electrolyte and electron transports, was confirmed by Li et al. The activation of the synthesized S, N co-doped graphene (NGS) by KOH allowed for the obtaining of a porous structure (ANSG), which introduced abundant structural defects into the carbon matrix. The ANSG catalyst (281 mV vs. RHE) showed a considerably lower overpotential at 10 mA cm^−2^ in 1 M KOH than NSG (452 mV vs. RHE). Similarly, significant differences were observed for electrochemical kinetics during the OER test; the Tafel slopes for ANGS and NGS were 108.9 and 172.1 mV dec^−1^, respectively [150].

One of the materials that has recently attracted significant attention in electrocatalytic applications is graphitic carbon nitride (g-C_3_N_4_), mostly due to its tailorable structure and high nitrogen content (pyridinic and pyrrolic N) in the carbon framework [168,169,170]. Nevertheless, the catalytic activity of bulk g-C_3_N_4_ suffers from low surface area and insufficient electronic conductivity. Doping with heteroatoms, designing nanostructures, and composite materials seem to be promising solutions for improving the electrocatalytic performance of carbon nitride. Wahab et al. reported the OER catalytic parameters for mesoporous g-C_3_N_4_ with high nitrogen content (48 wt.%); an onset potential of 1.51 V and a Tafel slope of 52.4 mV dec^−1^ in 1 M KOH, indicating favorable OER kinetics [78]. Furthermore, the high stability of the electrode was proven by the chronoamperometric response of mesoporous graphitic carbon nitride, which displays an attenuation of only 1.6% after 24 h. Ma et al. proposed the synthesis of a composite material consisting of self-assembly of g-C_3_N_4_ nanosheets and carbon nanotubes. The latter provided improved electron transfer and thus the catalyst generated a current density of 10 mA cm^−2^ at 1.60 V [72].

### 5.2. Carbon—Metal Hydroxides/Oxyhydroxides/Oxides

Metal oxide–hydroxide composites with carbons are being increasingly used to improve the electrocatalytic performance of the transition metal active phase. Such a combination promotes electron transport to the current collector by decreasing the thickness of the poorly conductive oxyhydroxide phase. In addition, the application of the porous carbons offers the benefits of enhanced access of the liquid phase reactants to the surface of the solid electrocatalyst. The formation of such composite materials enables an efficient way to reach the state-of-the-art OER activities (compare with Table 1). Representative recent reports are summarized below, with a special emphasis on the effects of carbon support on the transition metal phase reactivity. The characteristic parameters of the electrocatalysts are summarized in Table 3.

**Figure 10:**
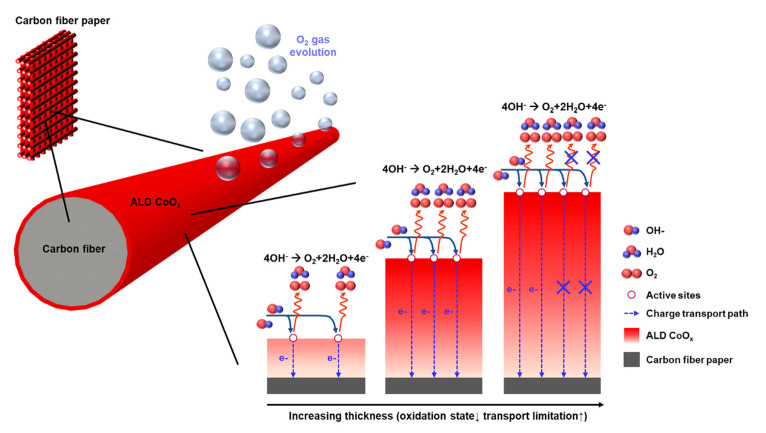
Schematic diagram of the change in oxidation state and charge transport length at various film thicknesses and the consequent OER activity. Reprinted with permission from Choi, H.J.; Han, G.D.; Bae, K.; Shim, J.H. Highly Active Oxygen Evolution on Carbon Fiber Paper Coated with Atomic-Layer-Deposited Cobalt Oxide. ACS Appl. Mater. Interfaces 2019, 11, 10,608–10,615. Copyright 2019, American Chemical Society [109].

Mixed Ni–Fe hydroxides were obtained through preferential templating on graphene edges where the edge-rich vertical graphene support provided the unoccupied density of states on the graphene edges, which are able to preferentially template specific valence orbital alignment of the Fe–O entities. This specific interaction of the graphene edge–metal component resulted in the formation of beneficial undersaturated and strained Fe-sites with high valence states, and at the same time, it boosted the formation of redox-activated Ni species. These two effects resulted in an improved OER reactivity [80]. For the Ni@Pt core–shell nanoplates and Ni/rGO composites, the overpotential of Ni/rGO is lower than pure Ni and the enhanced performance of Ni/rGO was ascribed to the possible effect of the rGO coatings. The GO was added in the synthesis of the Ni-Al LDH precursor and with the addition of hydrazine hydrate as a reducing agent in the reduction reaction, GO and Ni-Al LDH are simultaneously reduced. The morphology of obtained product is maintained as hexagonal nanoplates, which further improves the catalytic performance. According to the results of electrochemical impedance spectroscopy, the excellent conductivity of rGO plays a major role in reducing the influence of Ni oxidation in the process of water splitting, and it can effectively improve the electrical conductivity of the anode, facilitating electron extraction and shortening ion transport pathways during OER [100]. A uniformly coated carbon fiber paper with CoO_x_ layers was obtained by the atomic layer deposition technique (ALD). It was found that the oxygen evolution is not enhanced after a critical thickness of ~28 nm and the optimal thickness of the ALD-CoO_x_ film is dependent on two competing effects. The high oxidation state of cobalt ions in thicker CoO_x_ helps the oxygen evolution, whereas the introduction of a thick oxide coating decelerates the rate of charge transfer at the surface, which exemplifies the limitations of the oxide phases with respect to their low electrical conductivity (Figure 10). The larger Tafel slope for the thicker film could be a result of the inevitable charge transport limitation in the bulk region [109].

The excellent catalytic performance in both OER and ORR of N-rGO/NiCo-NiO-CoO and N-rGO/CoFe-CoFe_2_O_4_ obtained by deposition in an autoclave at 150 °C for 12 h was attributed to several factors. Firstly, it was supposed that the graphene matrix can enhance the conductivity and benefit the electron transfer during the catalytic process. Secondly, the nanoparticle with small and uniform size could provide sufficient active sites for electrocatalytic reaction. And finally, the existence of oxygen defects in the prepared composite can enhance the conductivity and accelerate the kinetics of surface redox reactions [96]. Subnanometer cobalt-hydroxide-anchored N-doped carbon nanotube forests, where abundant charge carriers in amorphous Co(OH)_x_ species, were found to trigger OER activity with a high current density and a low Tafel slope. The unique subnanometer scale morphology along with strong cobalt species-NCNT interaction is ascribed to an increase of the stability of the catalyst during prolonged repeated cycles [102]. One-step calcination of ternary NiFeMn-Prussian blue analogues/polymer hybrid precursors yielded a catalyst wherein a synergy between highly conductive continuous N-doped carbon network and oxidized metal species (Ni^2+^, Fe^2+^ and Mn^2+^) formed at the heterostructured Ni_0.36_Fe_0.64_/MnO_x_ interfaces enabled efficient withdrawing of electrons from OH^−^ and in turn charge transfer during OER [108]. In N-CNT/Ni–NiFe_2_O_4_ composites, the nanoparticles were anchored to the carbon nanotubes, thus greatly preventing agglomeration and achieving excellent structural and electrochemical stability. Moreover, both the N-CNT and the bimetallic spinel oxides contributed to the active sites not only for OER but also for HER and ORR [101]. Composites based on nanostructured iron-doped CoWO_4_ particles electrically linked to conductive carbon nanotubes, Co_0.5_Fe_0.5_WO_4_/CNT, were obtained using a facile one-pot hydrothermal method and exhibited overpotential at 10 mA cm^−2^ below 300 mV [97]. The OER performance may also be enhanced by combining multiple distinct oxide phases such as hollow Co_3_O_4_/CeO_2_ heterostructures and three-dimensional porous N-doped carbon nanofibers N-CNF networks [105]. In addition to graphene-based carbons and carbon nanotubes, other carbon materials are successfully used as supports for the oxide-hydroxide phase as in the case of Ni/NiO composite with N-doped activated carbon obtained from waste cauliflower leaves [92].

The successful electrocatalytic formulations present enhanced electron transport from the active phase to the current collector thanks to the application of well-conductive carbon-based component [96,97,100,109], which may also provide stabilization of the active metal hydroxides/oxyhydroxides/oxide phase. Various carbon structures are used, such as rGO or CNTs, usually doped with nitrogen to provide the desired electrical properties. Another common trait of the well-performing materials is the ability to stabilize high valence states of the TM cations in the active phase [80,108,109].

### 5.3. Carbon—Metal Chalcogenides

Transition metal chalcogenides (TMCs—sulfides, selenides, tellurides, where TM usually is Co, Ni, Fe), due to their multivalent oxidation states, abundant defects sites, and structural diversity, exhibit excellent abilities for electrocatalytic water splitting. Such materials are much cheaper than noble metals and exhibit noble-metal-like catalytic properties at the same time. Further, the electronic properties of TMCs can be tunable due to the ion-exchange process and the metal-chalcogen ratio variation in the electrocatalysts [178].

**Figure 11:**
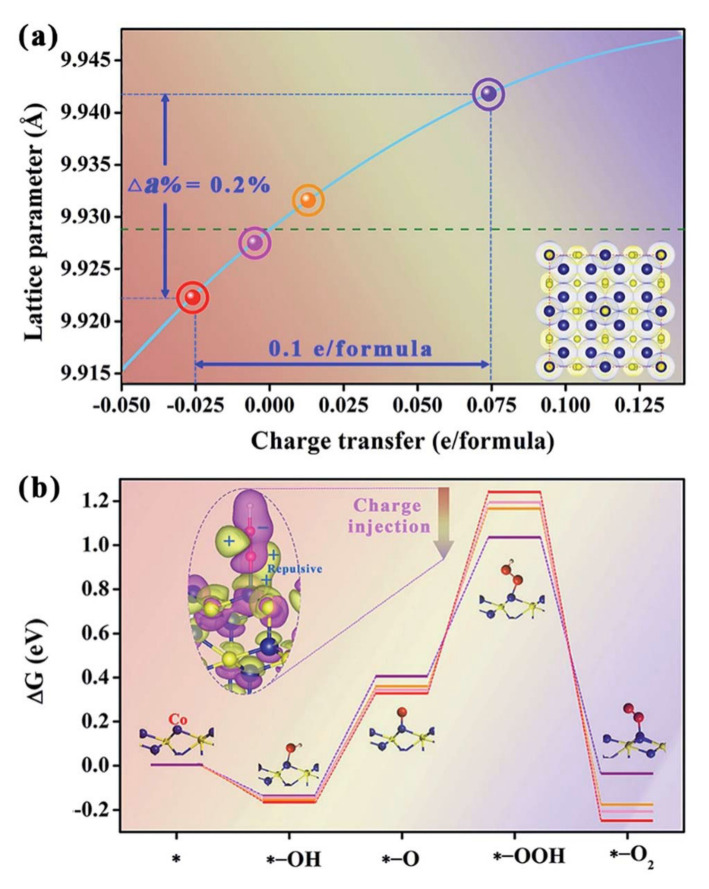
(**a**) DFT analysis of the dependence of lattice parameter of Co_9_S_8_ on the variation of injected charge transfer; (**b**) DFT calculated free energy landscape for the OER at 1.23 V vs. RHE (the standard potential for OER) on the Co_9_S_8_ (100) surface at different charging states. Inset in (**a**): structural model of the Co_9_S_8_ crystal; (**b**): absorption structure and charge density redistribution at different charging states (atoms with blue, yellow, red, and white colours represent Co, S, O and H atoms, respectively). Reproduced from Ref. [179] with permission from the Royal Society of Chemistry.

However, TMCs nanomaterials suffer from low stability, easy aggregation of particles, low electronic conductivity, and limited surface area, which can result in poor electrochemical activity. One of the strategies to overcome these obstacles aims at the fabrication of TMC/carbon composites and taking synergistic advantages of both materials. Combining TMCs with carbon materials significantly improves the intrinsic conductivity, facilitates charge transfer of the hybrids, as well as promotes the electroactivity of catalysts. To illustrate the effect of the electron interaction between a chalcogenide phase and the carbon support, density functional theory calculations were performed. It was found that a small increase in the lattice parameter of Co_9_S_8_, brought about by the different coupling to N-doped carbon, may result in a small but non-negligible charge injection to the sulfide phase, as summarized in Figure 11 [179]. The characteristic reactivity parameters of the carbon–metal chalcogenide electrocatalysts are summarized in Table 4.

Recently, great efforts have been taken to develop hybrid structures of carbons and transition metal sulfides, particularly cobalt-based ones [111,123,127,179,185,186,187]. Cao et al. fabricated Co_9_S_8_ embedded in a porous nitrogen-doped carbon matrix [187]. For example, a synthesized Co_9_S_8_/N-C composite reached an OER potential of 0.57 V at a current density of 5 mA cm^−2^ in 0.1 M KOH and had a Tafel slope of 75 mV dec^−1^. The chronoamperometry measurements revealed that after 55,000 s of continuous testing, Co_9_S_8_/N-C lost 6% of the initial current density, whereas a decrease of 70% in current density was observed for a RuO_2_/C (20% of an active phase) catalyst. This high electrocatalytic performance with sustained reactivity arises from covalent bonding between Co_9_S_8_ and N-doped carbon that facilitates the electron transfer. Also, the porous carbon matrix is favorable for the efficient transport of oxygen and electrolyte molecules during the OER processes. Ashok et al. designed a hierarchical Co/Co_9_S_8_/CNT nanostructure that required 1.5 V to achieve a current density of 10 mA cm^−2^ in 1 M KOH electrolyte with low Tafel slope (79 mV dec^−1^) [123]. After 20 h of the applied constant potential of 1.5 V, the current density was stable with minor variations.

Besides cobalt sulfides, Ni_7_S_6_ can be utilized as an OER catalyst, especially when coupled with nitrogen-doped graphene oxide [188]. Such a hierarchical porous nanocomposite (named by authors NGO/Ni_7_S_6_) has been proven to exhibit improved OER kinetic parameters, in particular a Tafel slope of only of 45.4 mV dec^−1^, which was lower by ~43.6 mV dec^−1^ than that for RuO_2_ measured under similar conditions in alkaline media. Similarly, Yang et al. reported a Tafel slope of 48 mV dec^−1^ for NiS@N/S-C catalyst. Hybrid material composed of NiS nanoparticles embedded in nitrogen/sulphur co-doped carbon matrix, derived from carbonization of metal–organic framework (MOF), exhibited an overpotential of 417 mV, which was delivered at a current density of 10 mA cm^−2^ in 1 M KOH. In addition to adequate OER activity, such composite catalyst exhibited satisfactory long-term durability for water splitting in alkaline media. During the chronopotentiometry measurements, the potential was stable (only insignificant voltage changes occurred) at 10 mA cm^−2^ for 10 h [184]. Le et al. fabricated P-doped iron sulfide nanowires integrated within a carbon matrix (P-Fe_7_S_8_@C) [189]. The electrocatalyst with the optimized amounts of P dopants exhibited remarkably low overpotential (210 mV) during OER tests at 20 mA cm^−2^ in 1 M KOH solution. The determined Tafel value was 42.5 mV dec^−1^, indicating high OER kinetic. 

Another approach consists of the fabrication of bimetallic sulfides [84,112,153,190,191,192]. For instance, NiCo_2_S_4_ combined with redox couples of Ni^3+^/Ni^2+^ and Co^3+^/Co^2+^ has been recently recognized as a highly efficient bifunctional oxygen electrocatalyst for rechargeable Zn–air batteries. He et al. synthesized nickel-cobalt sulfide nanoparticles confined in cages of carbon–nitrogen nanosheets (NiCo_2_S_4_/CNNs composites) [84]. The synergistic effect of both materials provided an overpotential of 360 mV at 10 mA cm^−2^) for oxygen evolution reaction in O_2_-saturated 0.1 M KOH. According to Han et al. NiCo_2_S_4_/N-doped CNTs showed excellent performance in the regenerative Zn–air system reaching the charge–discharge polarization of 0.63 V and a voltaic efficiency of ~67.2% [112]. Interestingly, after 150 cycles, the polarization decreased only by 0.06 V, reducing an energy efficiency by ~65.1%. As a comparison, standard commercial catalysts (Pt/C and RuO_2_) showed larger increments in the voltage gap after ~50 cycles, indicating their limited rechargeability. Jiang et al. proposed the fabrication of 3D Fe-Ni sulfide nanosheets/reduced graphene oxide catalysts, shortly named FeNiS_2_ NS/rGO, and compared OER activity of such material with RuO_2_ [192]. To reach a current density of 10 mA cm^−2^ in 1 M KOH, FeNiS_2_ NS/rGO and RuO_2_ required potentials of 1.43 V and 1.54 V, respectively. The measured Tafel slope for FeNiS_2_ NS/rGO was 40 mV dec^−1^, which was lower by 37 mV dec^−1^ than for RuO_2_. Furthermore, negligible variations in LSV curves for the freshly synthesized sulfide-based catalyst were measured before and after 5000 CV cycles in alkaline electrolyte, suggesting good stability of the catalyst in a long-term test. 

Besides sulfides, metal selenides coupled with the carbon matrix have recently been reported as active and stable OER catalysts. Ding et al. presented the catalytic parameters in OER reaction obtained for the hybrid material of CoSe_2_ nanoparticles with in-situ grown N-doped bamboo-like carbon nanotubes [180]. The CoSe_2_@N/C-CNT composite required an overpotential of 340 mV vs. RHE to reach a current density of 10 mA cm^−2^ in 1 M KOH, the calculated Tafel slope was 107 mV dec^−1^. For comparison, carbon nanotube-grafted core–shell structured CoSe_2_@C hybrids reported by Yuan et al. showed a slightly lower overpotential of 306 mV at 10 mA cm^−2^ in 1 M KOH and Tafel slope of 26 mV dec^−1^ [120]. Wang et al. compared the OER catalytic activity of the hybrid consisting of nickel selenide nanoparticles anchored on 2D multilayered graphitic carbon nitride (NiSe_2_/g-C_3_N_4_) with its monocomponent counterparts, namely NiSe_2_ nanoparticles (NPs) and g-C_3_N_4_ [113]_._ NiSe_2_/g-C_3_N_4_ composite exhibited lower potential and corresponding overpotential (1.52 V, 260 mV) than those of NiSe_2_ NPs (1.63 V, 400 mV) and g-C_3_N_4_ (1.77 V, 540 mV) at 40 mA cm^−2^. Tafel slopes for NiSe_2_/g-C_3_N_4_, NiSe_2_, and g-C_3_N_4_ were fitted as 143, 299, and 160 mV dec^−1^, respectively. Such catalytic parameters suggest faster charge transfer and favorable OER performance of NiSe_2_/g-C_3_N_4_ as well as point out the synergistic effect of the transition metal chalcogenide nanoparticle coupled with carbon matrix.

In a similar vein to sulfides, bimetallic and multimetallic selenides supported on carbons can generally achieve higher electrocatalytic efficiency during the OER process than monometallic selenides [114,117,118,122,126,182,193,194]. A mixed NiFe selenide supported by carbon fiber paper (NiFe-Se/CFP) required an overpotential of 281 mV at 10 mA cm^−2^ in alkaline media (1 M KOH), whereas such a value for FeSe_2_/CFP was 395 mV [118]. An enhanced electron-transfer kinetics was observed for NiFe-Se/CFP with a Tafel slope of 41 mV dec^−1^ (42 mV dec^−1^ lower than for FeSe_2_/CFP). Furthermore, the carbon-supported bimetallic chalcogenide catalyst has been proven to show good stability during the long-term (20 h) OER process. The post-test characterization of the NiFe-Se/CFP test suggests that the surface of the NiFe-Se nanoparticles undergoes a phase transformation from a bimetallic selenide phase to a mixed metal hydroxide/(oxy)hydroxide [118]. To understand the synergistic coupling between individual Co and Ni selenides, Liu et al. designed a hybrid catalyst consisting of heterostructured CoSe_2_/Ni_3_Se_4_ bimetallic selenides, N-doped carbon nanosheets, and Ketjen black carbon (CoSe_2_/Ni_3_Se_4_@NC/KB) [114]. To achieve a current density of 10 mA cm^−2^ in 1.0 M KOH, CoSe_2_/Ni_3_Se_4_@NC/KB needed 260 mV, which is lower by 180 and 120 mV than for CoSe_2_@NC/KB and Ni_3_Se_4_@NC/KB, respectively. At the same time, CoSe_2_/Ni_3_Se_4_@NC/KB displayed a Tafel slope of 68 mV cm^−1^, distinctly lower than those for the corresponding monometallic selenide-based composites. Chi et al. proposed synthesis of trimetallic Ni-Fe-Co selenides anchored on carbon fiber cloth as an efficient electrocatalyst (denoted as NiFeCoSe_x_/CFC) for OER in alkaline medium [126]. The synergistic interaction between trimetallic selenides and a selenide–carbon matrix leads to the lower overpotential of 150 mV at 10 mA cm^−2^ in OER tests in 1 M KOH with a Tafel slope of 85 mV dec^−1^, a greater double-layer capacitance of 200 mF cm^−2^, and much smaller charge transfer resistance than a corresponding unary/binary metal selenides.

Apart from transition metal sulfides and selenides, tellurides have also been reported as excellent materials in terms of OER electrocatalysis [181,183,195]. Wang et al. proposed synthesis of cobalt telluride encapsulated in N-doped CNT frameworks (CoTe_2_@NCNTFs), which exhibited an overpotential of 330 mV toward the OER (at 10 mA cm^−2^) in 1.0 M KOH electrolyte with a Tafel slope of 82.8 mV dec^−1^ [183]. Similar kinetic parameters were registered by Liu et al. for CoTe_2_ nanocrystals embedded in an N-doped graphitic carbon matrix (CoTe_2_@N-GC) [195]. Such a hybrid catalyst required an overpotential of 300 mV to deliver a current density of 10 mA cm^−2^; the value fitted Tafel slope was 90 mV dec^−1^. After driving a continuous OER test at a fixed potential of 1.55 V for 20 h, only negligible variations in current density over time were observed, pointing to the great stability of CoTe_2_@N-GC.

Sivanantham et al. compared OER activities in an alkaline electrolyte of core–shell cobalt–chalcogenide electrocatalysts with a thin carbon layer coating on each nanoparticle (Co_9_S_8_@NC, CoSe@NC, CoTe@NC) [124]. The nickel foam supported Co_9_S_8_@NC catalyst required an overpotential of 299 mV to deliver a current density of 10 mA cm^−2^ in 1 M KOH, which is 33 and 68 mV lower than those obtained for CoSe@NC and CoTe@NC composites, respectively. Moreover, the Tafel slope of Co_9_S_8_@NC was 65 mV dec^−1^, whereas for CoSe@NC and CoTe@NC calculated values were 76 and 112 dec^−1^, respectively.

### 5.4. Carbon—Metal Nitrides and Phosphides

**Figure 12:**
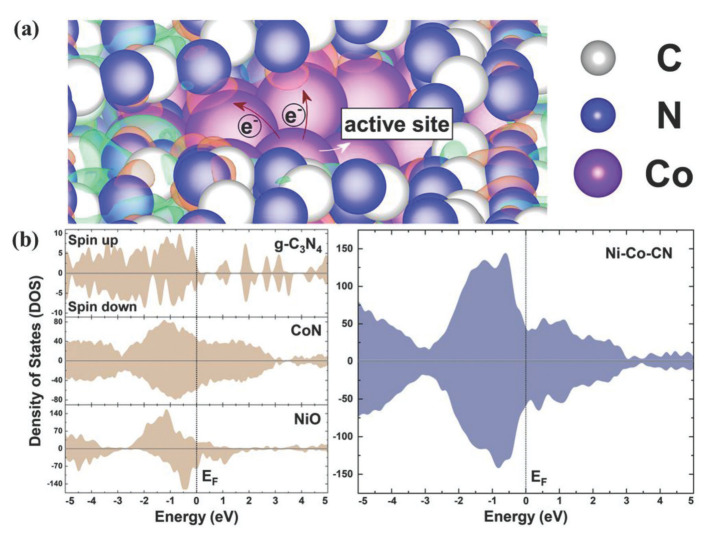
(**a**) Charge density difference of the Ni–Co–CN surface. Electron excess and deficiency are represented as red and green, respectively. The iso-surface was set as 0.0035 e Å^−1^. (**b**) Density of states (DOS) profiles of the partial (**left**) and ensemble (**right**) Ni–Co–CN species. Reproduced from Ref. [197] with permission from the Royal Society of Chemistry.

Other promising electrode materials for water splitting include transition-metal nitrides, in which the electrocatalytic activity, stability, and the number of active sites can be improved by controlling the composition, shape, and morphology of nanoparticles [90,134,135,136,137,138,144,145,146,149,152,196,197,198,199]. The presence of nitrogen atoms positively affects the electronic structure of the catalyst, leading to high electron density near the Fermi level and enhanced charge transfer. As illustrated in Figure 11, the strong interaction between g-C_3_N_4_ support and CoN may form a region of charge excess and enable electron transport from the cobalt active site to the neighboring Co–N bond [197]. Then, the electrons from the OER process could be accepted by the excess regions and accordingly improve the OER activity of the mixed material. To further study the electrical conductivities of all the systems, the densities of states (DOS) were calculated and are illustrated in Figure 12b. Among the samples, CoN shows the most outstanding conductivity, depicted by the highest DOS near the Fermi level, which leads to the excellent conductivity of Ni–Co–CN.

Furthermore, the intrinsic conductivity and OER activity of transition-metal nitrides can be modulated by merging the metal active phase with conductive supports such as carbons. Designing composite catalysts, namely transition-metal nitrides/carbon matrix, can also result in better accessibility of active sites and improved stability during electrochemical reactions due to avoiding the aggregation of nanoparticles and improving the corrosion resistance under strongly acidic and basic conditions. To get superior electrocatalytic activity and stability, the optimization of the carbon:TM is required. One of the approaches focuses on encapsulation of metal nanoparticles into an N-doped carbon shell, which may prevent the corrosion and agglomeration of metal NPs in an alkaline electrolyte. The characteristic reactivity parameters of the carbon–metal nitrides and phosphides electrocatalysts are summarized in Table 5.

Choi et al. reported electrochemical activity of composite catalysts consisings of mesoporous Co_3_N and amorphous N-doped carbon nanocubes (M-Co_3_N@AN-C) [149]. The catalyst reached the overpotential of 285 mV@10 mA cm^−2^ during OER measurements in 1 M KOH. Such excellent OER activity together with satisfactory stability results from 3-dimensional mesoporous structures that influence the surface area. Also, N-doped carbon layers improve the charge transfer ability and stability during the water-splitting process. The activity toward oxygen evolution reaction can even be boosted by designing multi-component nitrides, such as porous Co_3_FeN_x_/N-doped carbon nanoleaf arrays supported by carbon cloth (named Co_3_FeN_x_/NC LACC) [144]. Hierarchical structure and heterointerfaces between Co_4_N and FeN allowed for the reaching of a low overpotential of 270 mV at 10 mA cm^−2^ in 1 M KOH solution and the Tafel slope of 52.5 mV dec^−1^. Moreover, importantly for commercial electrocatalysts, the Co_3_FeN_x_/NC LACC exhibited outstanding durability during continuous tests, even after 40 h.

Recently, it has been revealed that the incorporation of foreign metal atoms into the crystal lattice of TM-based catalysts changes the local coordination environment and electronic configuration and hence improves the electrocatalytic performance. Lai et al. investigated the influence of S-doping of Ni_3_FeN compounds supported on N/S co-doped graphene, named S-Ni_3_FeN/NSG, on their electrocatalytic activity [152]. The catalyst required an overpotential of 260 mV to achieve a current density of 10 mA cm^−2^ in 1 M KOH. Such a value is lower by 40 and 67 mV than for Ni_3_FeN/NG and Ir/C, respectively. The corresponding Tafel slopes were 76, 100, and 110 mV dec^−1^ for S-Ni_3_FeN/NSG, Ir/C, and Ni_3_FeN/NG, respectively. The favorable catalytic reaction kinetics and improved OER activity of S-Ni_3_FeN/NSG can be attributed to the decoration of Ni_3_FeN nanoparticles by sulfur atoms. Quantum mechanical modelling pointed out that S-doping enhances the formation of -OOH* intermediates and noticeably decreases the maximum of change in Gibbs free energy for OER steps. 

Most recently, growing interest in terms of carbon-supported OER catalysts is related to transition-metal phosphides (TMPs), mostly due to their relatively low price and earth-abundance reserves [81,131,132,133,139,141,142,143,147,148,200,201,203,204,205,206,207,208,209,210]. The metal phosphides are thermodynamically less stable than corresponding metal hydroxides/oxides under oxidation potentials. Thus, gradually formed metal hydroxide/oxide phases on the TMPs surface during electrochemical reactions are considered to provide the actual active centers for oxygen evolution reaction, while the inner TMPs core acts as the conductive scaffold [61]. Since the morphology and/or structure of metal phosphides may undergo reconstruction upon a redox process, the TMPs do fall entirely under the conventional definition of the term *catalyst* [211]. Moreover, the transformations of composition and structure of the original catalysts into the final active species are difficult to predict since they may proceed along various paths. Therefore, different electrocatalysts should be extensively studied by experimental and theoretical methods to deeply understand their reaction mechanisms.

Yang et al. reported that cobalt phosphide nanoparticles dispersed within a nitrogen-doped carbon nanotube network (CP@NCNT) required an overpotential of 317 mV at 10 mA cm^−2^ with a Tafel slope of 75 mV dec^−1^ [201]. The achieved overpotential is 25 mV lower than for corresponding undoped composite material (bare CNTs as a carbon matrix), indicating that incorporation of nitrogen atoms promotes charge transfer and consequently enhances the catalytic activity. X-ray photoelectron spectroscopy analysis after 24 h of the amperometric *i-t* test revealed surface oxidation of cobalt phosphide nanoparticles proving that formed oxide species of CoOOH provides new active sites for OER. Tang et al. synthesized a bimetallic composite catalyst consists of Co_2_P nanoparticles coated with a nitrogen-doped carbon matrix and adsorbed at the surface of Fe_2_P microspheres [209]. A designed active NC−Fe_2_P interface ensured increased charge transfer rate of the catalyst and allowed the researchers to obtain outstanding catalytic parameters toward OER, such as an overpotential of 260 mV at a current density of 10 mA cm^−2^ in 1 M KOH, and a Tafel slope of 41 mV dec^−1^. Fe/Co-based oxyhydroxide species, formed during the OER process, led to excellent long-term stability. Most recently, the superior performance toward OER of iron-doped nickel–cobalt phosphide embedded in the amorphous carbon layer has been reported [147]. The hybrid material (called Fe–NiCoP@C) reached an overpotential of 270 mV (10 mA cm^−2^, 1 M KOH), which is lower by 49 mV than for NiCoP. To get insight into electrode reaction kinetics, electrochemical impedance spectroscopy was conducted. An electrolyte resistance for Fe–NiCoP@C and NiCoP were 3.2 Ω and 4.2 Ω, respectively, indicating low internal and interfacial resistances between the catalyst surface and the electrolyte for Fe–NiCoP@C. Furthermore, DFT calculations showed that iron dopant affects the electrical property of the composite catalyst as well as plays a role as the active site for the OER process and reduces the free energies of adsorption of the O-containing intermediates. 

## 6. Interfaces in OER Electrocatalysts

The main interface encountered with the OER electrocatalysts is the solid–liquid–gas one, where the material meets the electrolyte and the reaction product, oxygen. Other interfaces include phase boundaries between components of the composite material and phase boundaries created between outermost layers of materials (e.g., oxides, phosphides) and the oxidized/hydroxylated surfaces directly responsible for the catalytic action. The optimization and rational design of three-phase interfaces are of paramount importance in electrocatalytic applications and include the exposure of the active sites, mass diffusion enhancement, and boosting of electron transfer [10]. Improvement in one direction may hinder the overall performance due to a decreased efficiency of another. 

At the interfaces between two active components, reconstructed active centers exhibit different chemical properties due to modified bonding and electronic interactions with the neighboring atoms. Moreover, in the vicinity of the interface, materials exhibit structural disorder, increased defects concentration, additional phases, and various molecular components. Therefore, the new active centers located at or in the vicinity of the interfaces can be responsible for the actual catalytic activity and stability rather than the original components [212,213]. Typical interfaces present in the electrocatalysts include hydroxide-oxide composites, such as Ni(OH)_2_–CeO_2_, where the improved catalytic activity can be assigned to the strong electronic coupling of the oxide and hydroxide phases, which favorably modulates the interaction between the reaction intermediate and catalyst [214]. Another type involves an in situ formation of the active phase overlayer, especially from materials unstable under reaction conditions, as in β-NiO(OH)/Ni_2_P@NiO catalyst supported on Mg_2_O(OH)_2_-like phase, which is formed during the oxygen evolution reaction, where the Ni_2_P nanoparticles are immediately oxidized forming a core–shell structure. The enhanced performance of the core–shell material is hypothesized to result from the reactivity modulation due to the development of a lattice strain in the interfacial area, and the high surface area of the supported electrocatalyst [215]. 

Nanocomposites with enhanced electrical conductivities can be created by combining two or more different types of carbon materials. For example, developed for energy storage application, a composite of carbon nanofibers (CNF) with natural graphite microspheres was found to form a continuous conductive network resulting in improved cycling performance. Moreover, improved penetration of the composite surface by the electrolyte resulted in a faster electrode reaction kinetics and the CNF/graphite network provided mechanical stabilization for the electroactive system by accommodating volume changes and thus preventing its mechanical degradation by cracking [67].

An accurate design, synthesis, modification and detailed characterization of well-defined carbon–carbon or carbon–non carbon materials’ interfaces are the basis for the engineering of nanocomposites with tailored properties targeted for specific applications. Every step of the composite preparation will influence the final electrocatalytic activity, notably, heteroatom doping of carbon materials, precursor types of active components, and applied synthetic procedures [213]. The approaches to study composited materials usually lie between two borderline cases. On one hand, a deep understanding of the complex interfacial structures and the interaction of the comprising materials is studied. On the other hand, the preparation of model composites with regulated and optimized interface structure is pursued to gain more fundamental knowledge. Eventually, both approaches mix and should lead to the same goal, which is an improvement in the performance of the electrocatalytic materials.

Interface engineering in carbon-based nanocomposites employs two main types of chemical interactions: covalent and non-covalent. Both types can result in the desired interfacial interactions, however, different synthesis strategies are used for the preparation of the materials. Covalent interactions (chemical bonding) are most often obtained through techniques such as chemical vapor deposition, condensation reaction, radical polymerization, and hydrothermal techniques. Non-covalent interactions, typically achieved by self-assembly mixing and in situ polymerization approaches, usually involve π–π interactions, hydrogen bonding, and van der Waals forces [67].

A brief overview of the recent developments in the heterointerfaces in OER electrocatalysts is presented here. For the Ni_2_P/Fe_2_P–O system, an occurrence of synergistic effects of Ni_2_P(O)/Fe_2_P(O) interfaces, formed by active surface layers and metallic phosphide bulk, is proposed. On the surface, phosphates possibly act as a proton-coupled electron transfer mediator, while doped oxyhydroxides could accelerate the formation of O–O bonds and lower the activation barrier for OER. The underlying Ni_2_P/Fe_2_P could retain its conductive properties and promote the electron transfer process [216]. A heterojunction within hierarchical CoP-nanowire–FeP-nanorod branched heterostructures endows it with intimate interfacial contact between CoP and FeP, thus inducing a built-in electric field at the interface, which is said to promote the charge transfer and enhancement of the electrocatalytic activity [217]. In the hierarchical Co(OH)_2_/Ag/FeP hybrid structure, where the Ag nanoparticles were uniformly distributed on the surface of FeP nanorod arrays, and the latter decorated Co(OH)_2_ nanosheets, which provided an increased catalytically active area, resulted in a changed electron structure due to the formation of interfaces involved in Co(OH)_2_/Ag/FeP hybrid, which is considered to be beneficial to generate low-charge state Fe^2+^ and highly-oxidized Co^3+/4+^ [218]. A heterostructure resulting from a direct combination of CoP and CeO_2_ mainly benefits from the abundant interfaces between CoP nanosheets and CeO_2_ nanoparticles that generate a high concentration of oxygen vacancies and catalytically active sites, as well as tuning the surface electronic states [219]. Another direct combination of phosphorus-rich and oxygen-rich phases, cobalt hydroxide–black phosphorus nanosheets, resulted in an interface that exhibits small interlayer distance, large interlayer binding energy, and significant charge transfer between Co(OH)_2_ and black phosphorus, indicating a strong interlayer interaction and metallic interface, resulting in an improved conductivity and fast electron transfer [220]. 

Sulfide active phases are often investigated for both OER and HER, with potential application as bifunctional catalysts. Introducing Ni(OH)_2_ to the Ni_3_S_2_ yielded an electrocatalyst with low ionic and electronic resistances enabled by modulation of the surface atomic configuration of the sulfide phase. This led not only to high OER activity but also accelerated the Volmer step and OH– adsorption during the HER. Moreover, the obtained loose and interconnected nanoforest-like architecture facilitated the full exposure of active sites with enhanced electrolyte infiltration and bubble escape [221]. Strongly coupled NiS nanoparticles/Bi_2_WO_6_ nanosheets formed a 0D/2D heterojunction catalyst with interfacial synergistic effect and a strongly coupled electronic effect. The NiS nanoparticles were evenly distributed on the surface of Bi_2_WO_6_ nanosheets allowing for maximum atom utilization. The re-coordinated nickel and oxygen species act as bridges at the interface, resulting in a novel valence-bond environment of the O–Ni–S bond structure to enhance the charge and mass transfer for the surface reactions [222]. Cu_2_S–CoO_x_/Cu foam profited from the synergistic effect between Cu_2_S and CoO_x_, and electronic coupling between the two components. The catalyst benefits also from the distribution of CoO_x_ on Cu_2_S nanowires, which possess good electronic conductivity [223].

The formation of an intimate hydroxide–oxide interface is similarly beneficial for OER. In particular, the addition of CeO_2_ to hydroxide phases appears to result in strong electronic interaction formed by the intimate hydroxide–CeO_2_ interfaces, which might favorably modulate the interaction between intermediate and catalyst [214]. The CeO_2_ addition effect results probably from the enrichment of the electronic distribution near the Fermi level, boosting the electron transfer efficiency from local active centers to adsorbates [224]. Other combinations are also reported, such as dual-interface structure in NiV-LDH@FeOOH on Ni foam, which brings about improved electrical conductivity and optimized electronic structure [225].

In carbon-based composites, the carbon component provides a high surface area to deposit the active phase or support and active phase. A combination of Ni_2_P nanoparticles and Mg_2_O(OH)_2_-like support grown on carbon paper results in immediate oxidation of the Ni_2_P nanoparticles during OER, through the core–shell structuring, eventually forming active and stable β-NiO(OH)/Ni_2_P@NiO catalyst supported on a Mg_2_O(OH)_2_-like phase. The authors suggest that the high performance of the catalyst results from the development of a substantial degree of lattice strain in the core–shell nanoparticles and the realization of the supported catalyst with a high surface area [215]. Formation of carbon-supported OER catalyst by decomposition of precursors based on the metal–organic framework compounds, like FeHP-ZIF-67, may lead to a durable connection between the carbon and phosphide components, which persists after the oxidation of the phosphide to oxyhydroxide species. These kinds of materials demonstrate increased resistance to aggregation of Co_2_P nanoparticles, while the formation of active interfaces by partially connecting the Fe_2_P microspheres is also observed, which facilitates a rapid charge transfer [209]. N-NiMoO_4_/NiS_2_ catalyst on carbon fiber cloth exhibits enhanced electron transfer from N-NiMoO_4_ to NiS_2_ through abundant epitaxial heterogeneous interfaces at the atomic level. Moreover, this superaerophobic binder-free 3D open electrode with 2D nanosheets provides a large specific surface area that favors gas release from the surface (H_2_ and O_2_) [226].

## 7. Conclusions and Outlook

The search for active and stable materials for the oxygen evolution reaction has become a field with enormous interest for the development of efficient and cost-effective electrochemical devices like water electrolyzers or aqueous metal–air batteries. In this review, we provide the most recent investigations on the incorporation of carbon-based composite materials and analyze the most relevant advances in activity and stability issues. As a general trend, the use of carbon phases in the OER electrocatalyst formulation aids in increasing the density of active sites, allowing for smaller metallic particles as well as better accessibility of reactants to the catalyst surface.

**Figure 13:**
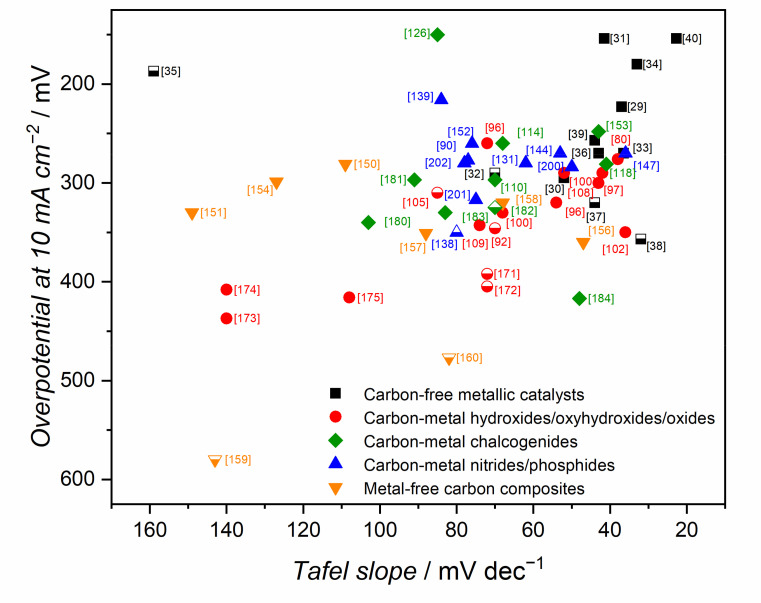
Graphical summary of the best reported electrocatalysts for oxygen evolution reaction divided into different classes of carbon-based composite materials; presented data are for OER process in 1 M (full symbol) and 0.1 M (half-empty symbol) alkaline solution.

Carbon-based electrocatalysts were classified in this review according to their composition into four categories: (i) metal-free carbon composites (ii) composites of carbon with metal hydroxides/oxyhydroxides/oxides, (iii) composites of carbon with metal nitrides/phosphides, and (iv) carbon–metal chalcogenides. The graphical summary of the best electrocatalysts reported in this review following the above classification is presented in Figure 13. To date, the lowest OER overpotential and lowest Tafel slope are achieved with carbon-free metal catalysts, with Fe and Ni oxyhydroxides and phosphides presenting the most promising activity within noble metal-free catalysts in an alkaline environment. One of the main problems arising with these materials is that the electrical conductivity of oxide phases could not be improved sufficiently, which causes ohmic resistance and hinders electron transfer within the catalyst layer for practical electrodes, which are usually different from lab-scale ideal conditions.

Carbon–metal oxides and carbon–metal nitrides appear as promising formulations combined with graphitic and other resistant carbon substrates for the OER. The activity for carbon–metal catalysts falls in the range of about 250–350 mV overpotential and the Tafel slope varies between 40 and 80 mV dec^−1^, with a few exceptions presenting either Tafel or overpotential falling behind those numbers. Meanwhile, metal-free catalysts still present the highest overpotential and Tafel slope values, suggesting that the presence of metallic species is required for proper activity at the current state of knowledge.

Advances in recent years have led to significant improvements in the activity and stability of carbon-based composites and future perspectives are good in terms of the further enhancement of performance by following this strategy. The main positive effects are related to increased catalyst surface area, improved electrical conductivity, and synergistic effects between carbon and metallic sites for enhanced intrinsic activity compared to single phases. However, more effort must be dedicated to the durability and scalability of catalysts. The oxidation of carbon appears as a drawback for the practical application of carbon-based composites that need further and deeper investigation. Experiments in full electrolyzer mode are also required to demonstrate the feasibility of this technology with this kind of OER composite catalyst. All this, in parallel with the development of improved anion exchange membranes, will boost the implementation of AEM water electrolyzers for the economic production of hydrogen.

## Figures and Tables

**Table 1:** Comparison of the OER performance of the best representative electrocatalysts in alkaline electrolytes.

Electrocatalyst	KOH Concentration	Substrate	Loading/mg cm^−2^	Overpotential at 10 mA cm^−2^/mV	Tafel Slope/mV dec^−1^	Ref.
**G-FeCoW**	1 M	GCE *	0.21	223	37	[29]
**α-Co4Fe(OH)_x_**	1 M	GCE	0.28	295	52	[30]
**(Ni,Fe)OOH**	1 M	NF *	4.0	154	41.5	[31]
**Co_3_O_4_C-NA**	0.1 M	Cu foil	0.2	290	70	[32]
**CoO-MoO_2_**	1 M	NF	N/A	270	36.5	[33]
**Core–shell NiFeCu**	1 M	NF	10.2	180	33	[34]
**Ni_3_S_2_ nanorods**	0.1 M	NF	37	187	159	[35]
**Fe_7_S_8_ nanosheets**	1 M	GCE	0.143	270	43	[36]
**CoSe_2_ nanosheet**	0.1 M	GCE	0.142	320	44	[37]
**CoTe_2_ nanofleeces**	0.1 M	GCE	0.25	357	32	[38]
**Co_4_N nanowire**	1 M	carbon cloth	0.82	257	44	[39]
**FeP/Ni_2_P**	1 M	NF	8	154	22.7	[40]

* GCE—glassy carbon electrode; NF—nickel foam.

**Table 2:** Selection-comparison of metal-free carbon electrocatalysts.

Catalyst	Electrolyte	Overpotential at 10 mA cm^−2^/mV	Tafel Slope/mV dec^−1^	Ref.
ANGS (activated S, N co-doped graphene)	1 M KOH	281	109	[150]
SNG@GF (N and S-doped graphene on graphite foam)	1 M KOH	330	149	[151]
GNP (N, P, and O-doped carbon)	1 M KOH	299	127	[154]
N-GRW (N-doped graphene nanoribbons)	1 M KOH	360	47	[156]
PAN-CCC (N-doped cotton cloth)	1 M NaOH	351	88	[157]
NMWNT (N-doped multi-walled carbon nanotubes)	1 M NaOH	320	68	[158]
G-BNG (stacked nanofilm of graphene on B,N-codoped graphene)	0.1 M KOH	580	143	[159]
OCC (oxidized carbon cloth)	0.1 M KOH	477	82	[160]

**Table 3:** Selection-comparison of carbon—metal hydroxides/oxyhydroxides/oxides.

Catalyst	KOH Electrolyte Concentration	Overpotential at 10 mA cm^−2^/mV	Tafel Slope/mV dec^−1^	Ref.
Ni-Fe Hydroxide/edge-rich vertical graphene	1 M	276	38	[80]
Ni/NiO/N-doped activated carbon	0.1 M	346	70	[92]
N-rGO/NiCo-NiO-CoO	1 M	260	72	[96]
N-rGO/CoFe-CoFe_2_O_4_	1 M	320	54	[96]
Co_0.5_Fe_0.5_WO_4_/CNT	1 M	290	42	[97]
Ni nanoplates/rGO	1 M	330	68	[100]
Ni@Pt core–shell nanoplates/rGO	1 M	290	52	[100]
Ni-NiFe_2_O_4_/N-CNT	1 M	340	51 *	[101]
Co(OH)_x_/N-CNT	1 M	350	36	[102]
Hollow Co_3_O_4_/CeO_2_-heterostructure/N-doped carbon nanofibers	0.1 M	310	85	[105]
Ni_0.36_Fe_0.64_/MnOx/N-doped graphitic carbon, Mn/Ni = 0.2	1 M	300	43	[108]
CoO_x_/carbon fiber paper	1 M	343	74	[109]
CoO_x_/NrGO	0.1 M	392	72	[171]
TaO_x_/CNF	0.1 M	405	72	[172]
CoO-Co/CNF	1 M	437	140	[173]
NiCo-loaded CNF	1 M	408	140	[174]
Co_3_O_4_/CNF	1 M	416	108	[175]
NiCo_2_O_4_/CNF	6 M	223	174	[176]
FeCo_2_O_4_/CNF	6 M	130	100	[177]

* with Ni foam.

**Table 4:** Selection-comparison of carbon–metal chalcogenide electrocatalysts.

Catalyst	KOH Electrolyte Concentration	Overpotential at 10 mA cm^−2^/mV	Tafel Slope/mV dec^−1^	Ref.
MoS_2_ wrapped N-doped carbon-coated Co nanospheres	1 M	297	70	[110]
CoSe_2_/Ni_3_Se_4_@N-doped carbon nanosheets/ketjen black carbon	1 M	260	68	[114]
NiFe-Se/carbon fiber paper	1 M	281	41	[118]
NiFeCoSe_x_/carbon fiber cloth	1 M	150	85	[126]
Fe_x_Ni_1−x_S_2_/C	1 M	248	43	[153]
CoSe_2_@N-doped bamboo-like carbon nanotubes	1 M	340	103	[180]
Fe-Co_1.11_Te_2_@N-doped carbon nanotube	1 M	297	91	[181]
NiSe-Ni_3_Se_2_/MWCNT	0.1 M	325	70	[182]
CoTe_2_ encapsulated in N-doped carbon nanotube frameworks	1 M	330	83	[183]
NiS@N/S-C	1 M	417	48	[184]

**Table 5:** Selection-comparison of carbon–metal nitrides and phosphides electrocatalysts.

Catalyst	KOH Electrolyte Concentration	Overpotential at 10 mA cm^−2^/mV	Tafel Slope/mV dec^−1^	Ref.
Ni_3_N/B-doped graphene oxide	1 M	280	78	[90]
Ni_12_P_5_ nanosheets coupled with oxidized MWCNTs	1 M	280	62	[131]
Co_5.47_N@N-doped rGO	0.1 M	350	80	[138]
NiFeP@ N-doped carbon sponge	1 M	216	84	[139]
Co_3_FeN_x_/N-doped carbon nanoleaf arrays@carbon cloth	1 M	270	53	[144]
Fe–NiCoP embedded in the amorphous carbon layer	1 M	270	36	[147]
S-Ni_3_FeN/N,S co-doped grephene	1 M	260	76	[152]
NiCo_2_P_x_/CNTs	1 M	284	50	[200]
CoP@N-doped carbon nanotube network	1 M	317	75	[201]
FeNi_3_@N-doped carbon	1 M	277	77	[202]
